# Supplementary figures and images for: Single-cell transcriptomics of the human parasite Schistosoma mansoni first intra-molluscan stage reveals tentative tegumental and stem-cell regulators
Source: Sci Rep. 2024 Mar 12;14:5974. doi: 10.1038/s41598-024-55790-3 (PMC10933418; doi:10.1038/s41598-024-55790-3)

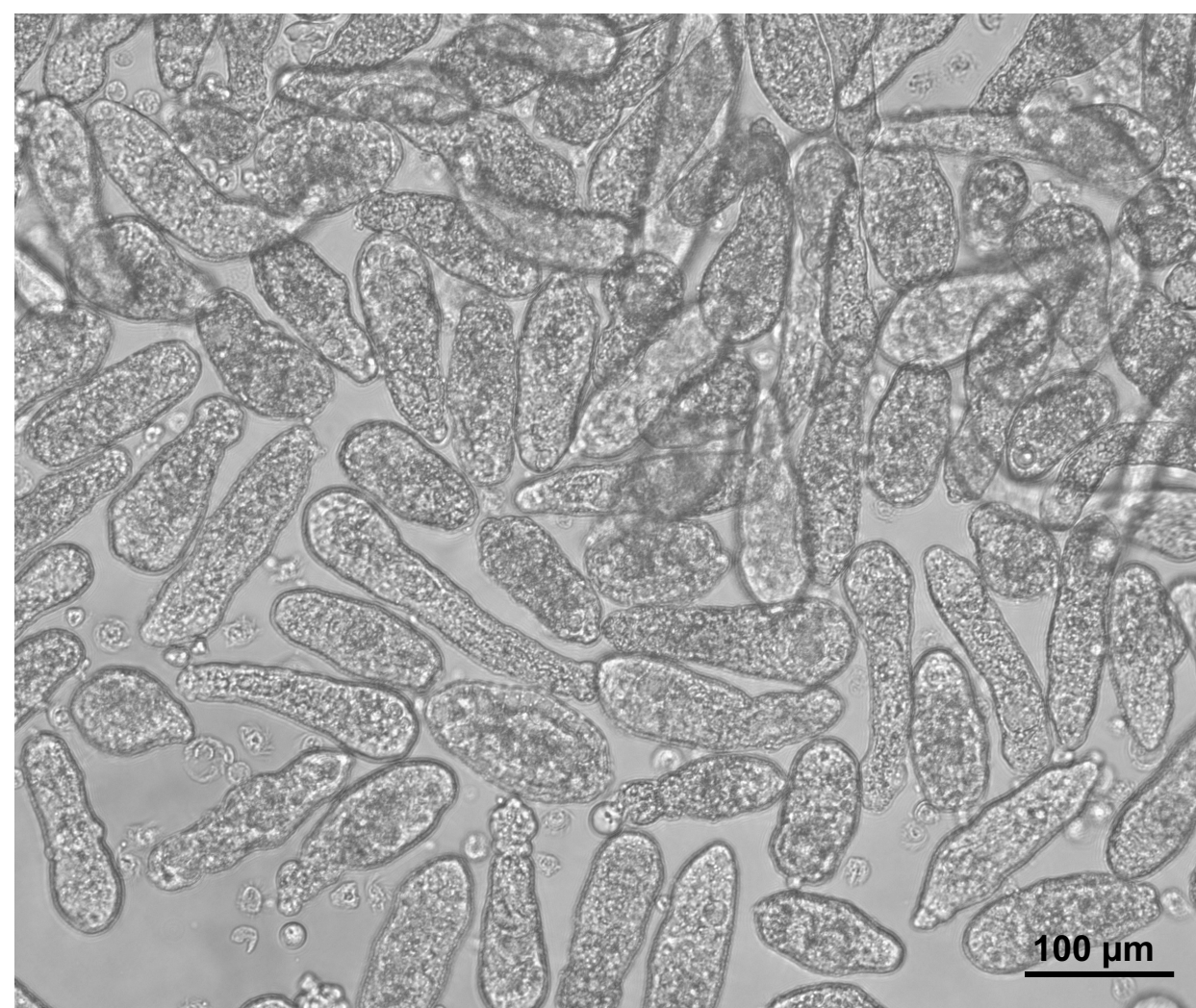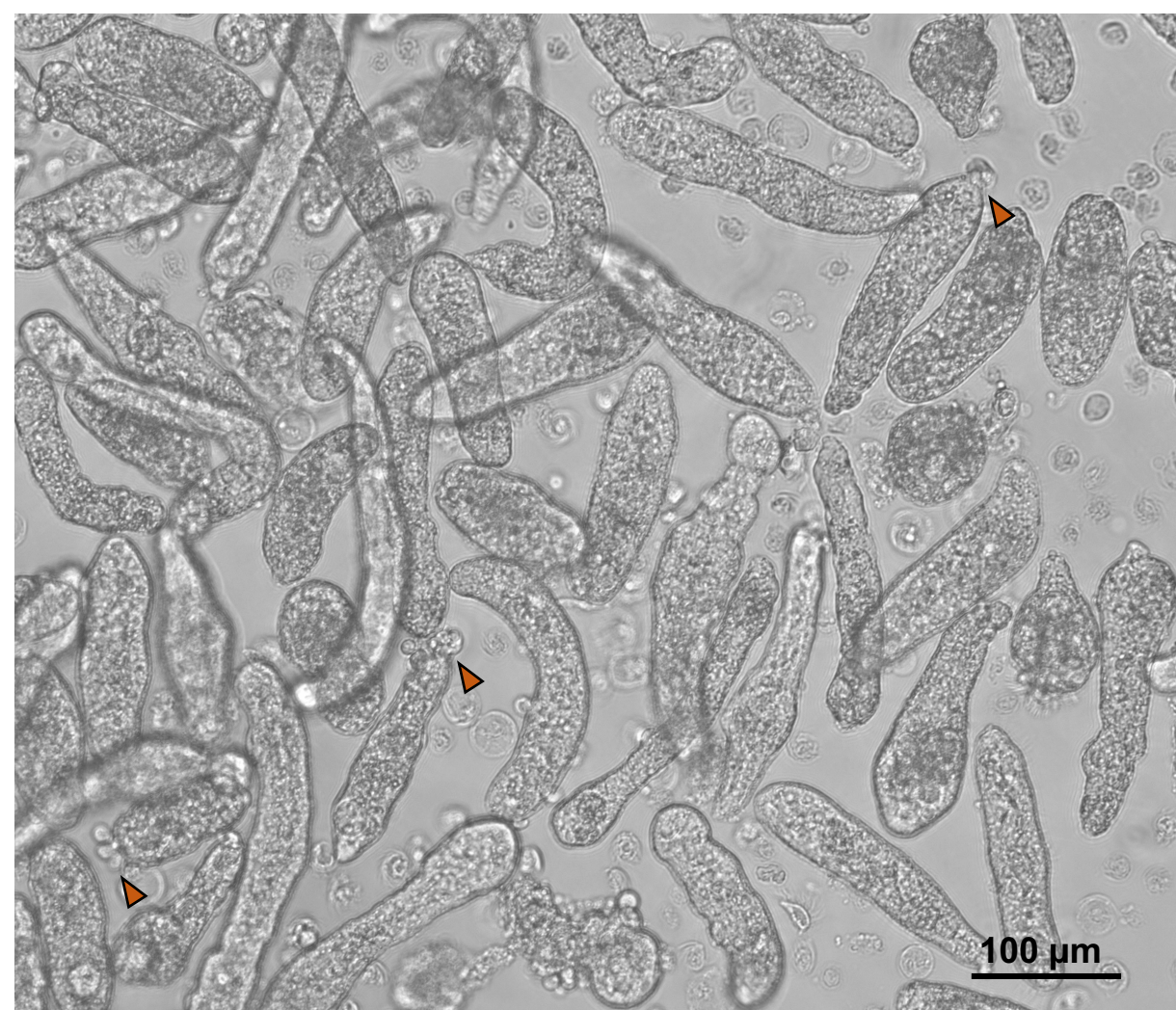

Supplement: Supplementary file 2 — Supplementary Information 2. [file 41598_2024_55790_MOESM2_ESM.pdf]

**A**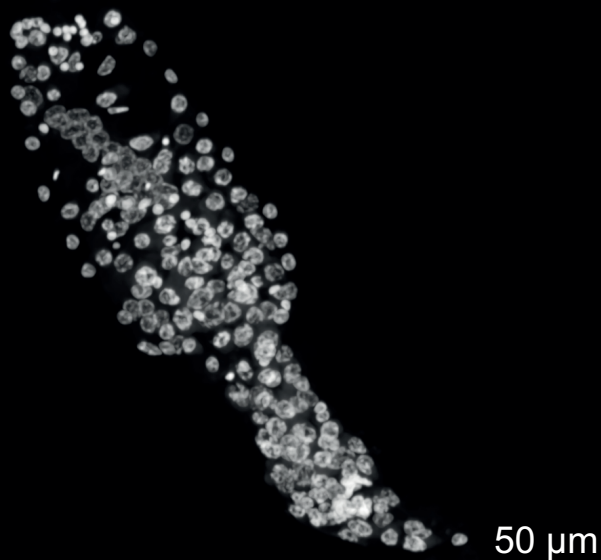**B**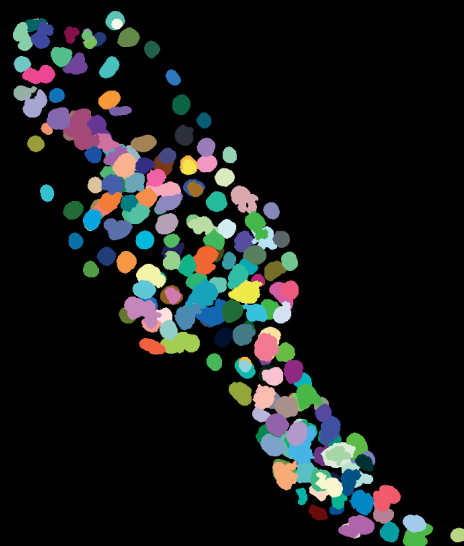**C**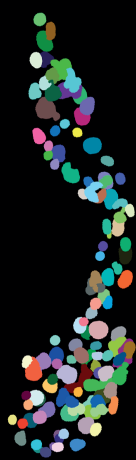

Flatworm 1  
118 nuclei

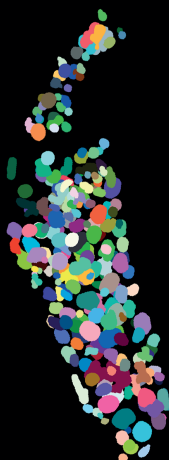

Flatworm 2  
254 nuclei

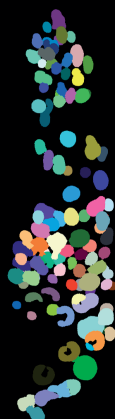

Flatworm 3  
112 nuclei

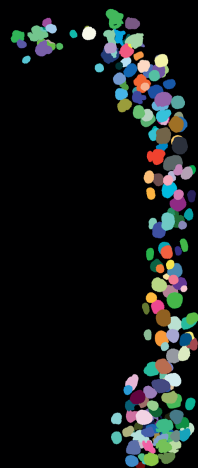

Flatworm 4  
182 nuclei

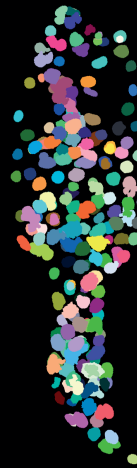

Flatworm 5  
177 nuclei

Supplement: Supplementary file 3 — Supplementary Information 3. [file 41598_2024_55790_MOESM3_ESM.pdf]

# Sm5HTR (Smp\_126730)

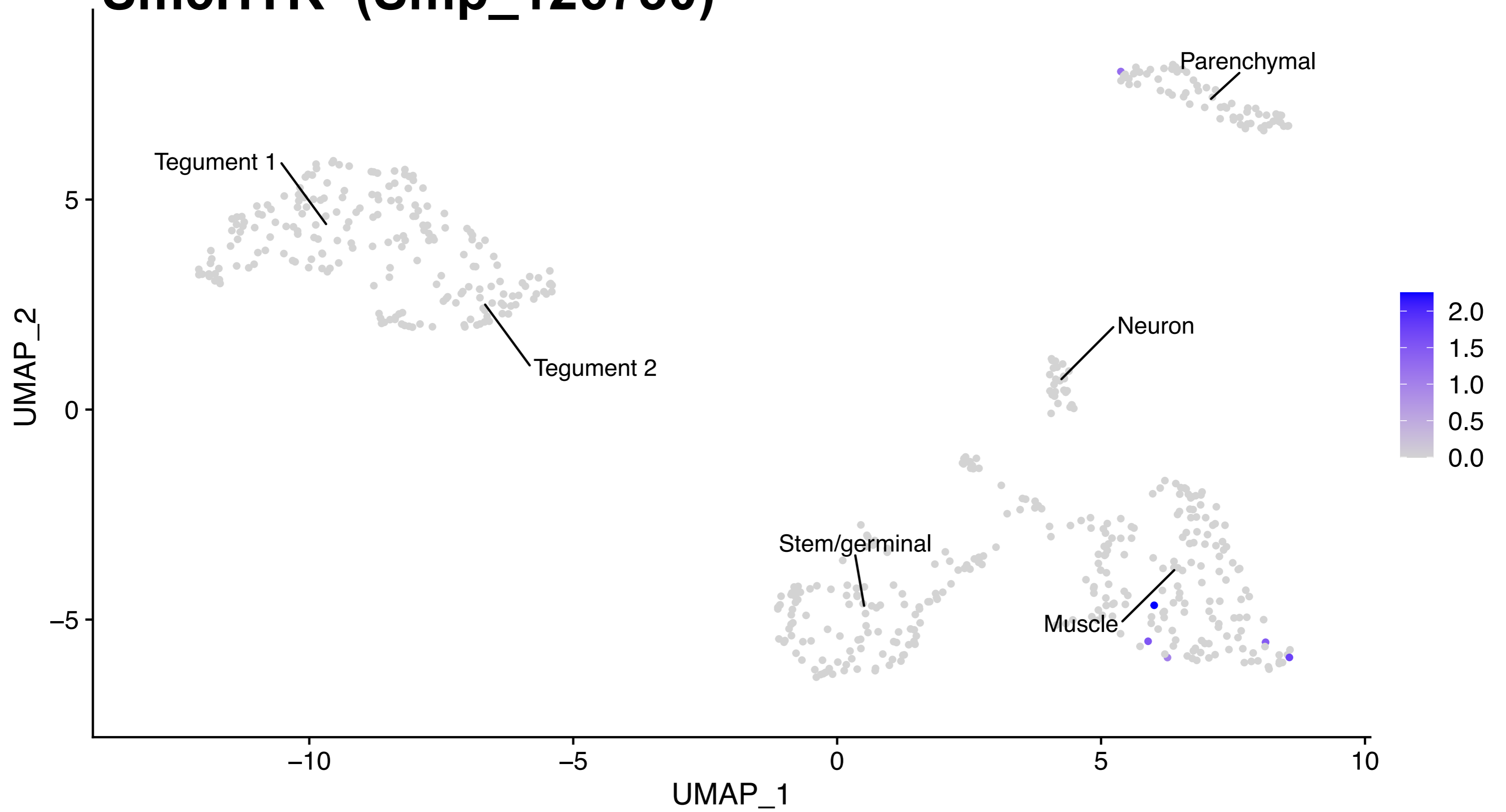

# \*Smp\_245850

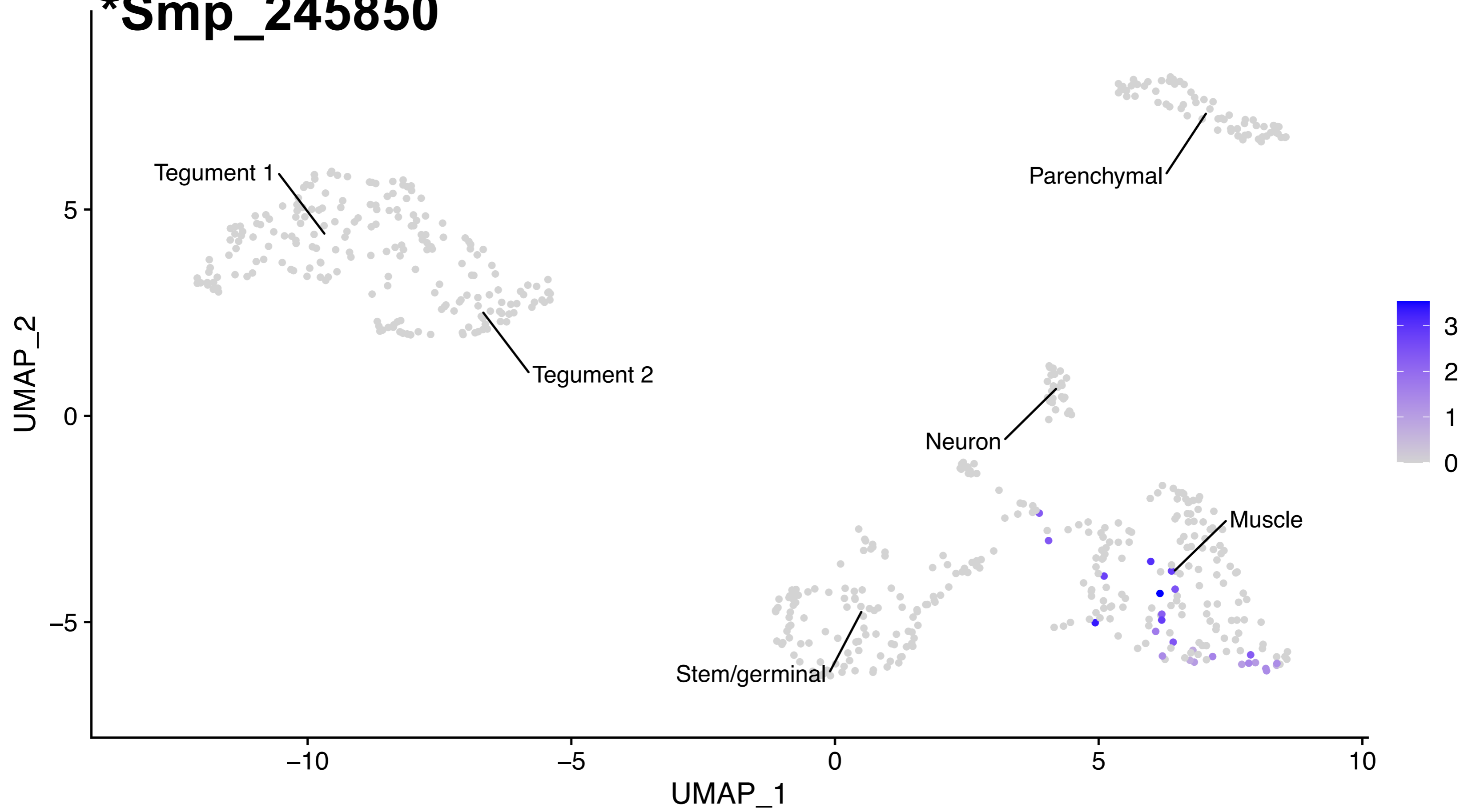

Supplement: Supplementary file 4 — Supplementary Information 4. [file 41598_2024_55790_MOESM4_ESM.pdf]

**A**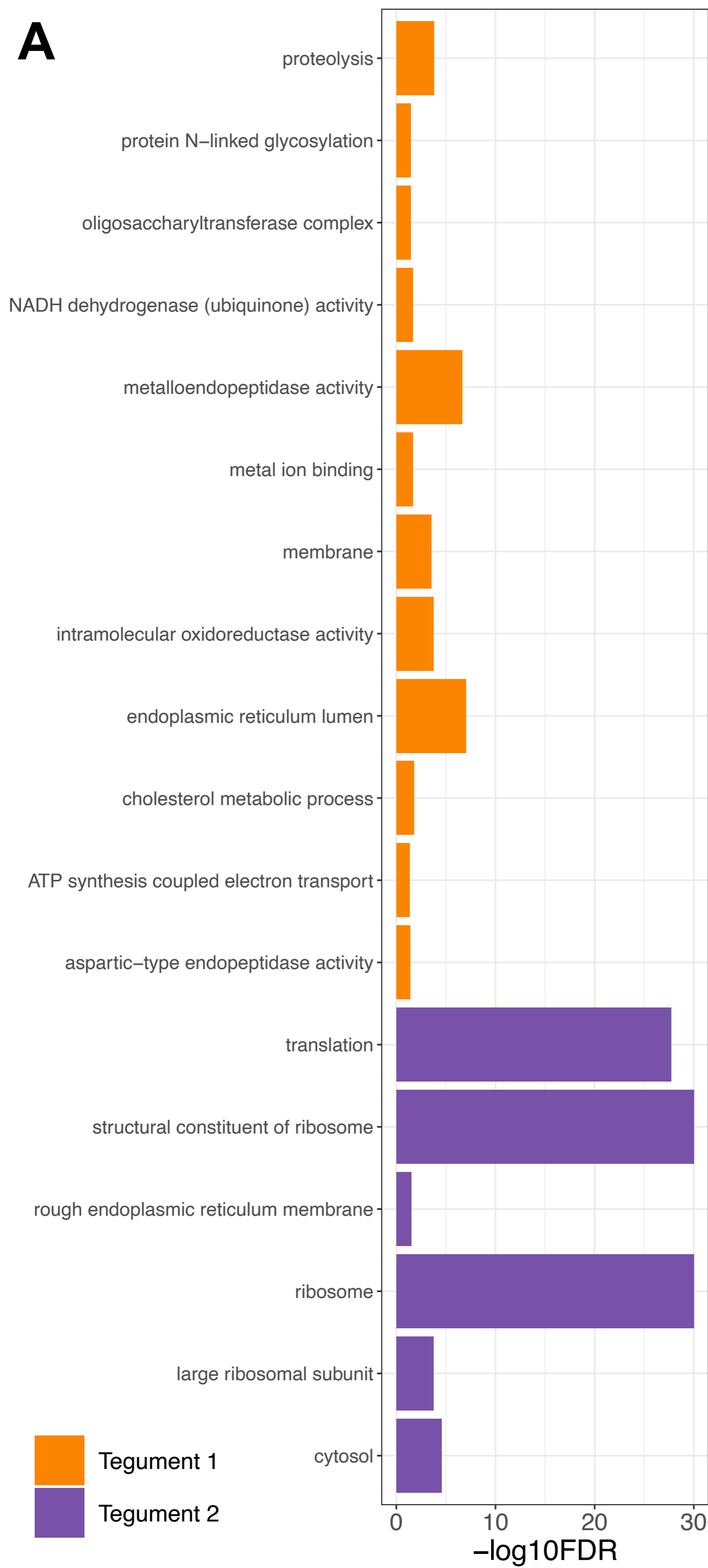**B**

### Plexin domain-containing protein (Smp\_348500)

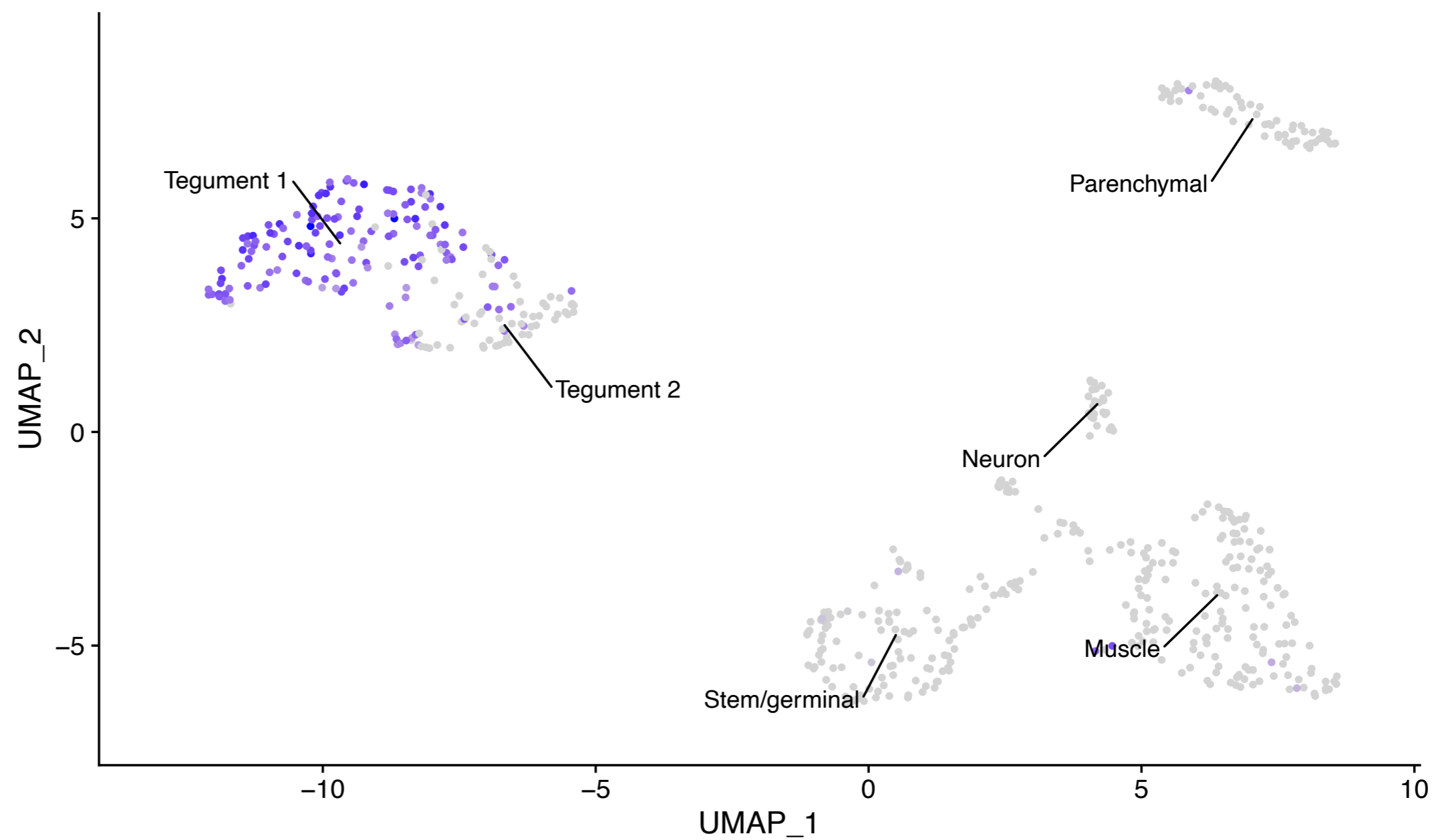

### Arrestin\_C domain-containing protein (Smp\_121950)

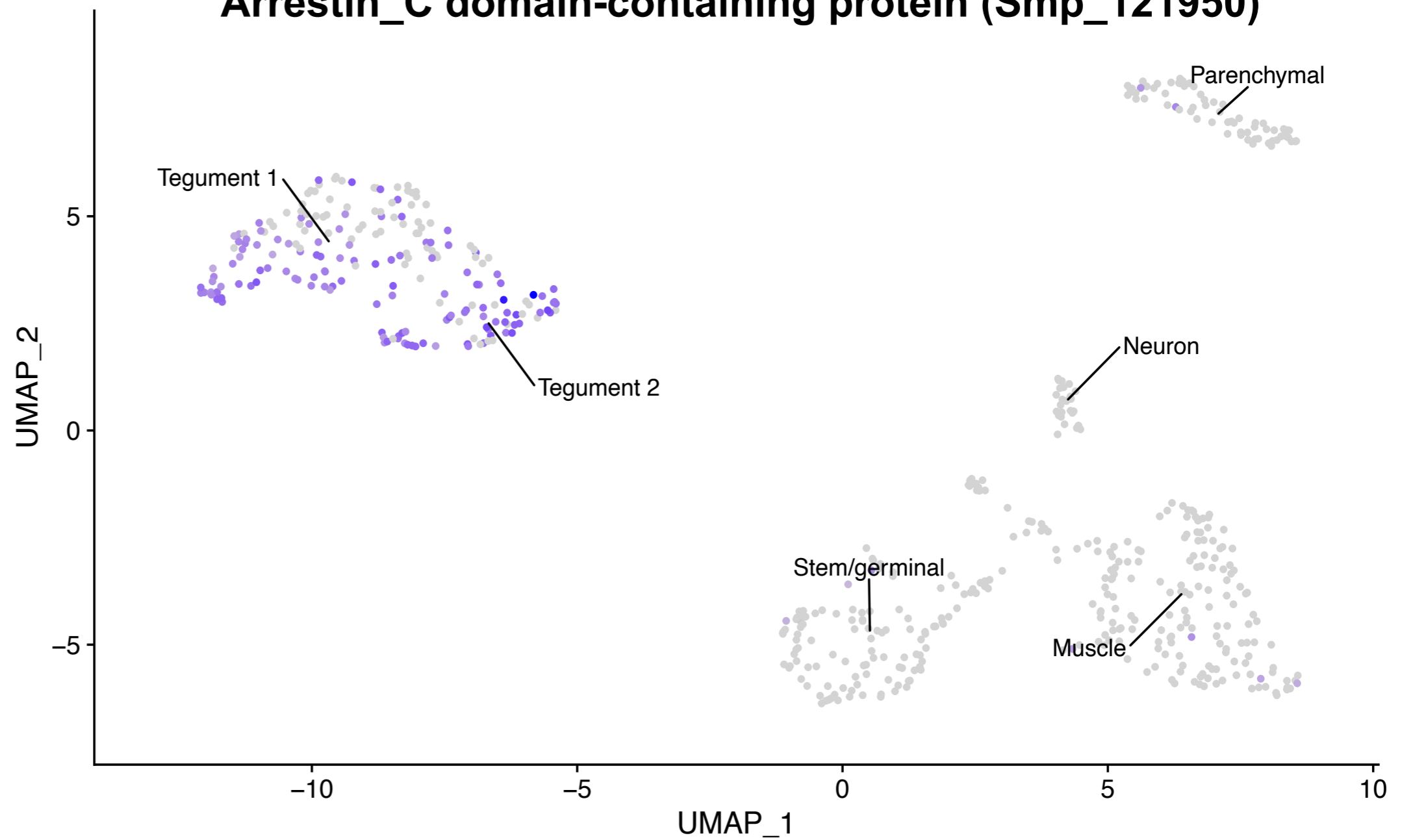

Supplement: Supplementary file 5 — Supplementary Information 5. [file 41598_2024_55790_MOESM5_ESM.pdf]

Biological processes in the tegument of *S. mansoni* developmental stages

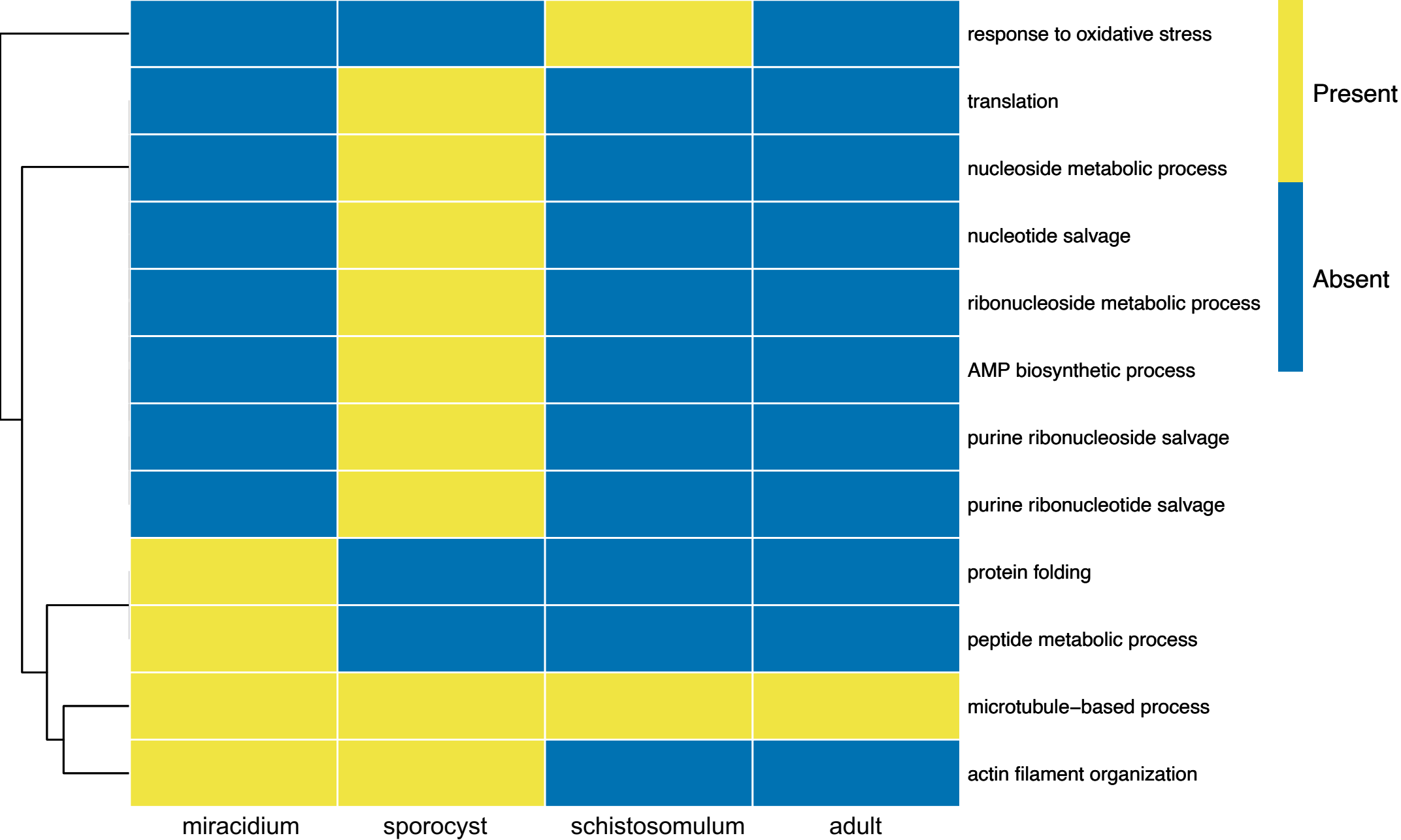

Supplement: Supplementary file 6 — Supplementary Information 6. [file 41598_2024_55790_MOESM6_ESM.pdf]

TOP 30 marker genes for Tegument clusters in indicated *S. mansoni* developmental stages

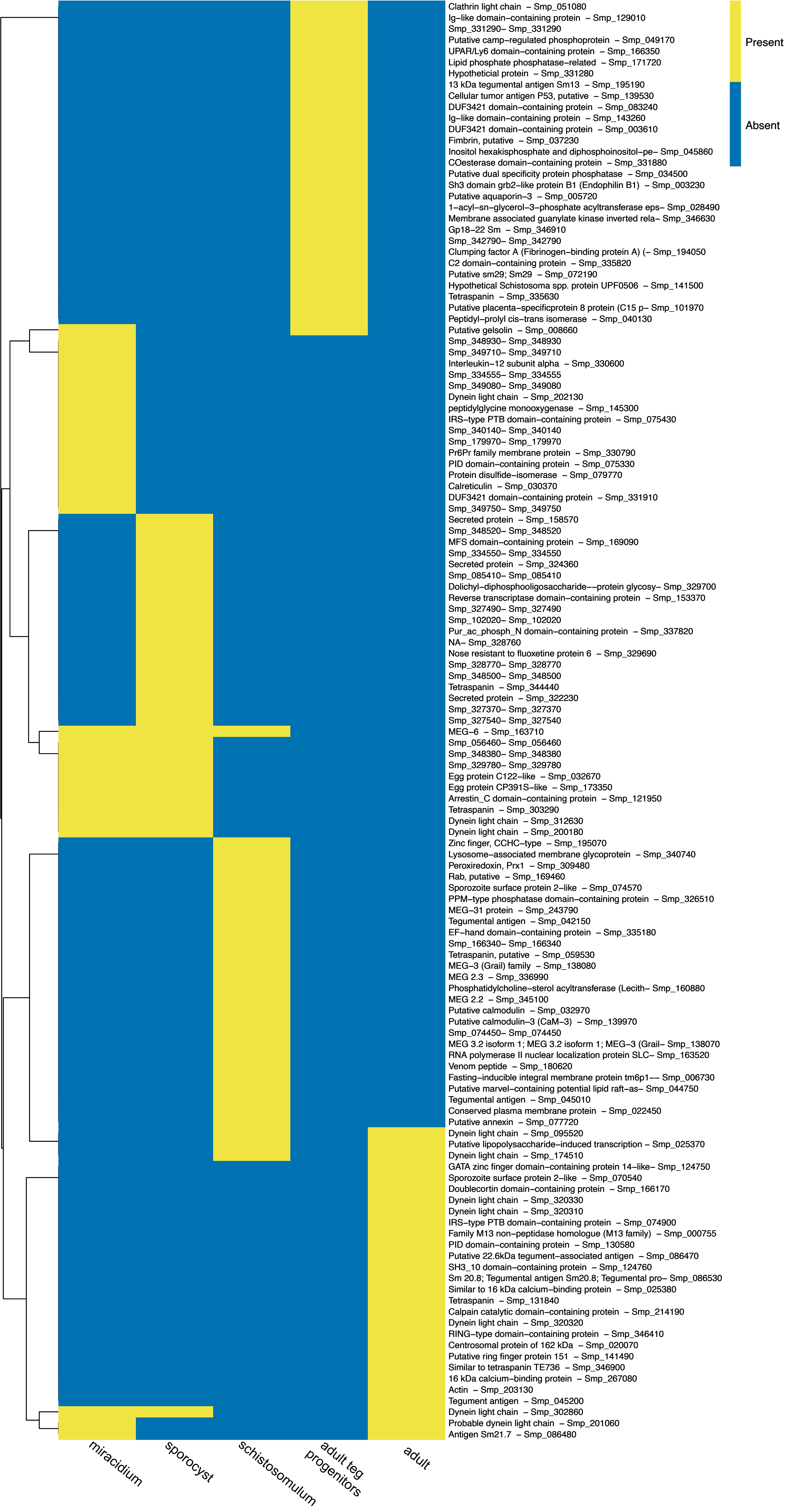

Supplement: Supplementary file 7 — Supplementary Information 7. [file 41598_2024_55790_MOESM7_ESM.pdf]

Biological processes in the Stem cell clusters of *S. mansoni* developmental stages

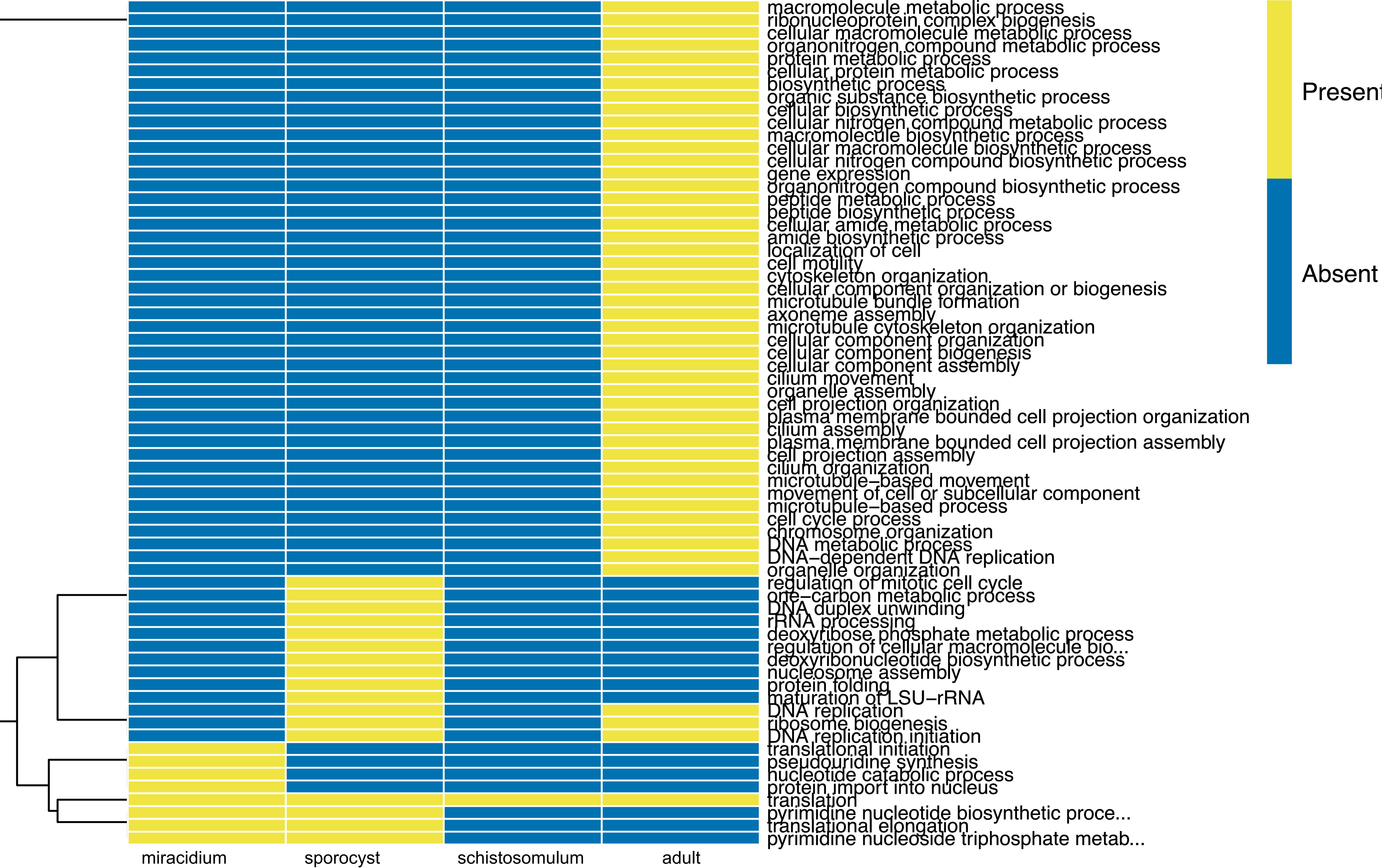

Supplement: Supplementary file 8 — Supplementary Information 8. [file 41598_2024_55790_MOESM8_ESM.pdf]

TOP 30 marker genes for Stem cells in indicated *S. mansoni* developmental stages

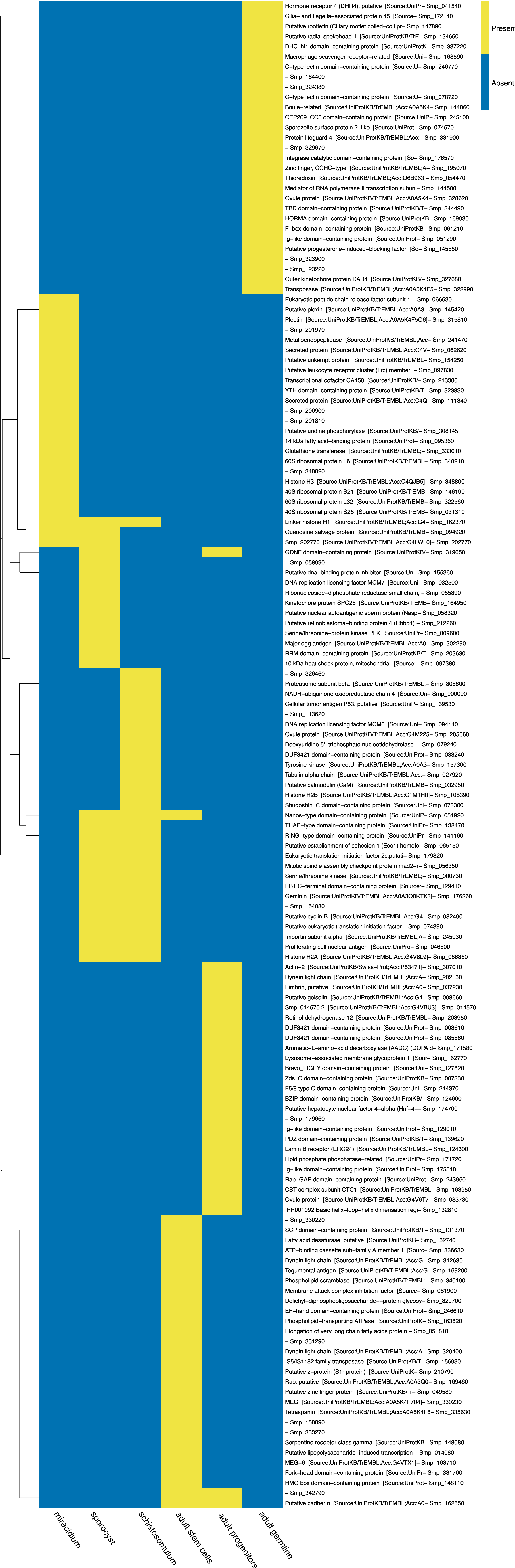

Supplement: Supplementary file 9 — Supplementary Information 9. [file 41598_2024_55790_MOESM9_ESM.pdf]

Biological processes in the Parenchyma cells of *S. mansoni* developmental stages

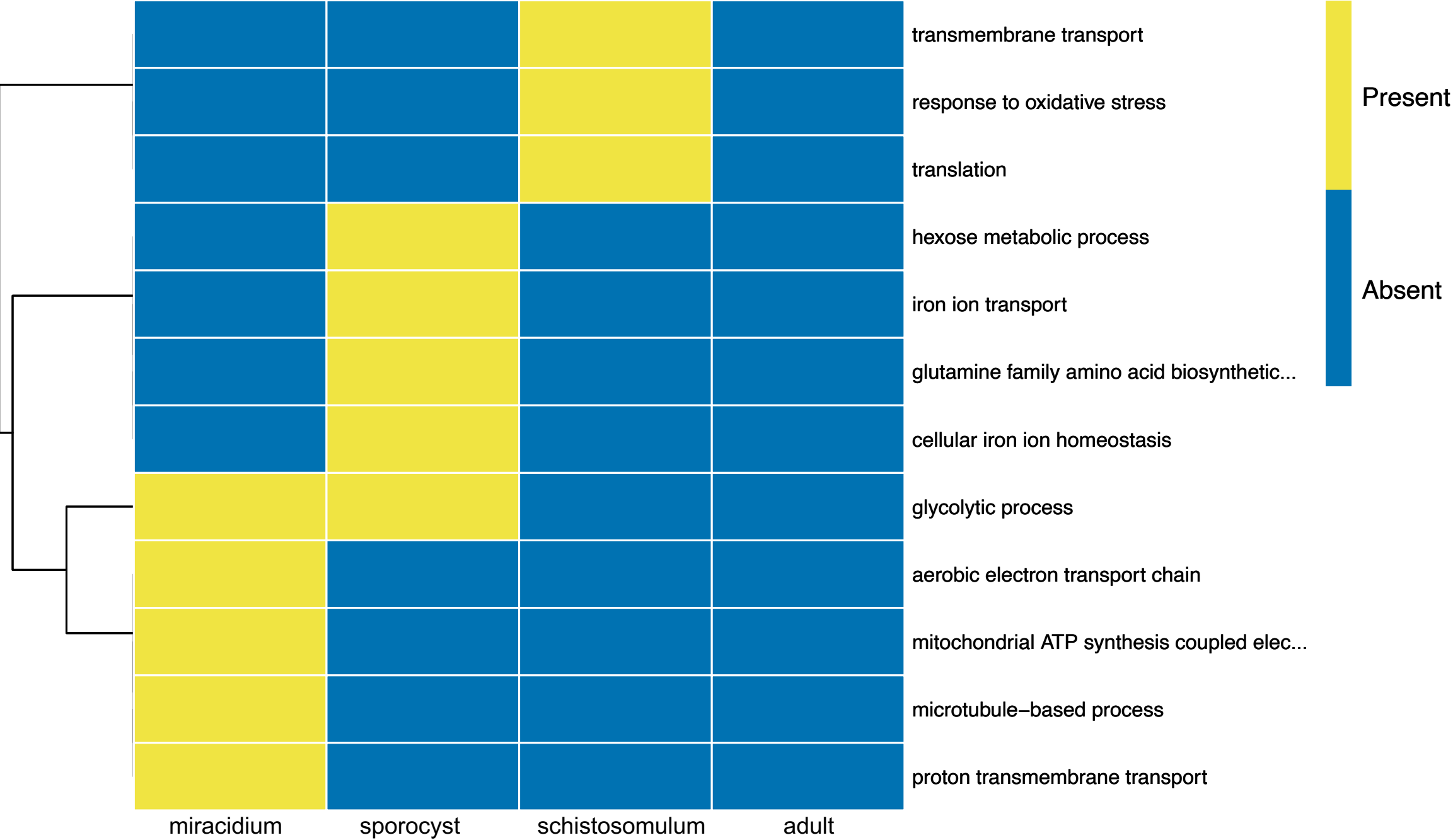

Supplement: Supplementary file 10 — Supplementary Information 10. [file 41598_2024_55790_MOESM10_ESM.pdf]

TOP 30 marker genes for Parenchyma cells in indicated *S. mansoni* developmental stages

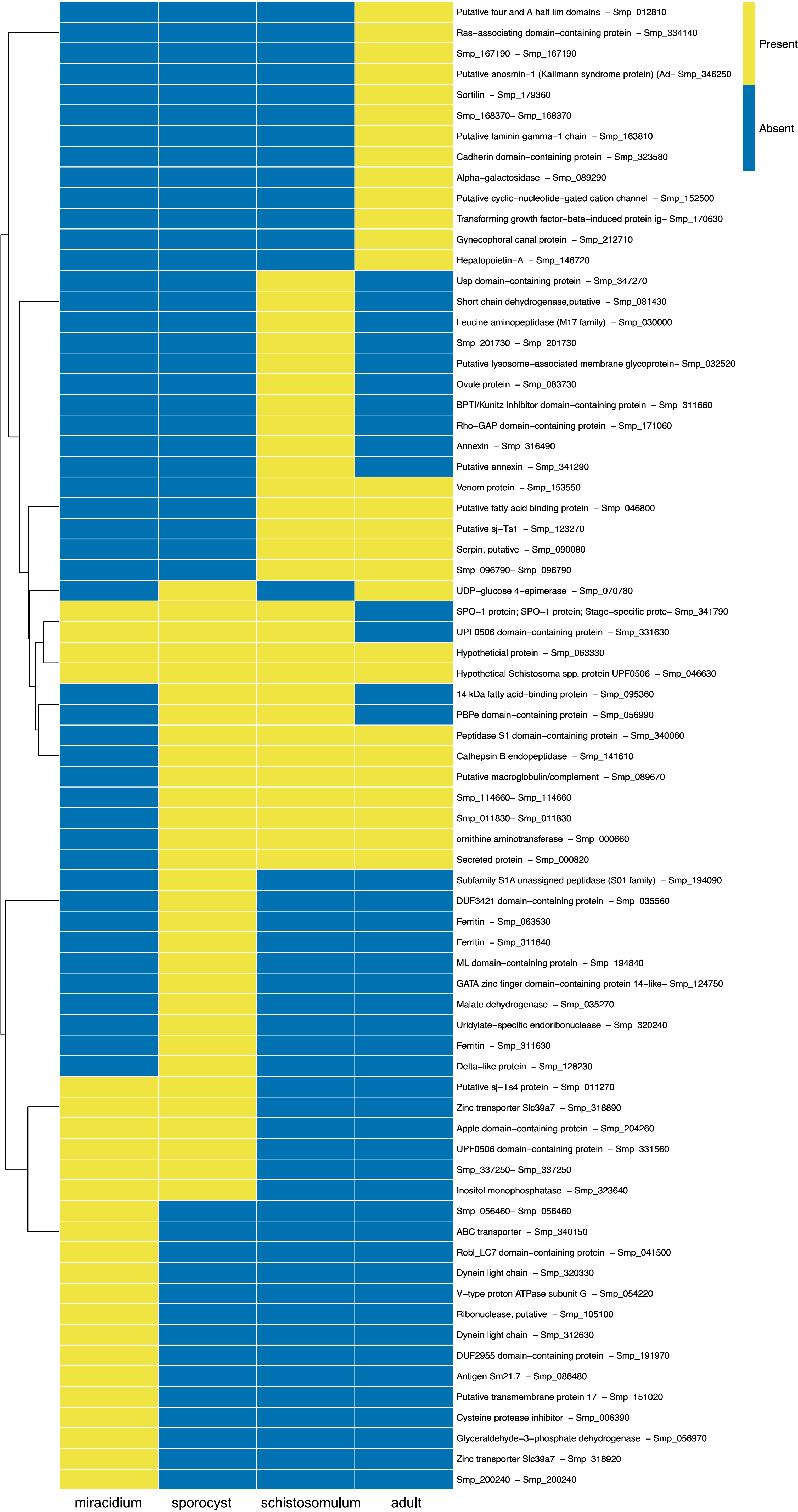

Supplement: Supplementary file 11 — Supplementary Information 11. [file 41598_2024_55790_MOESM11_ESM.pdf]

Biological processes in Muscle cells of *S. mansoni* developmental stages

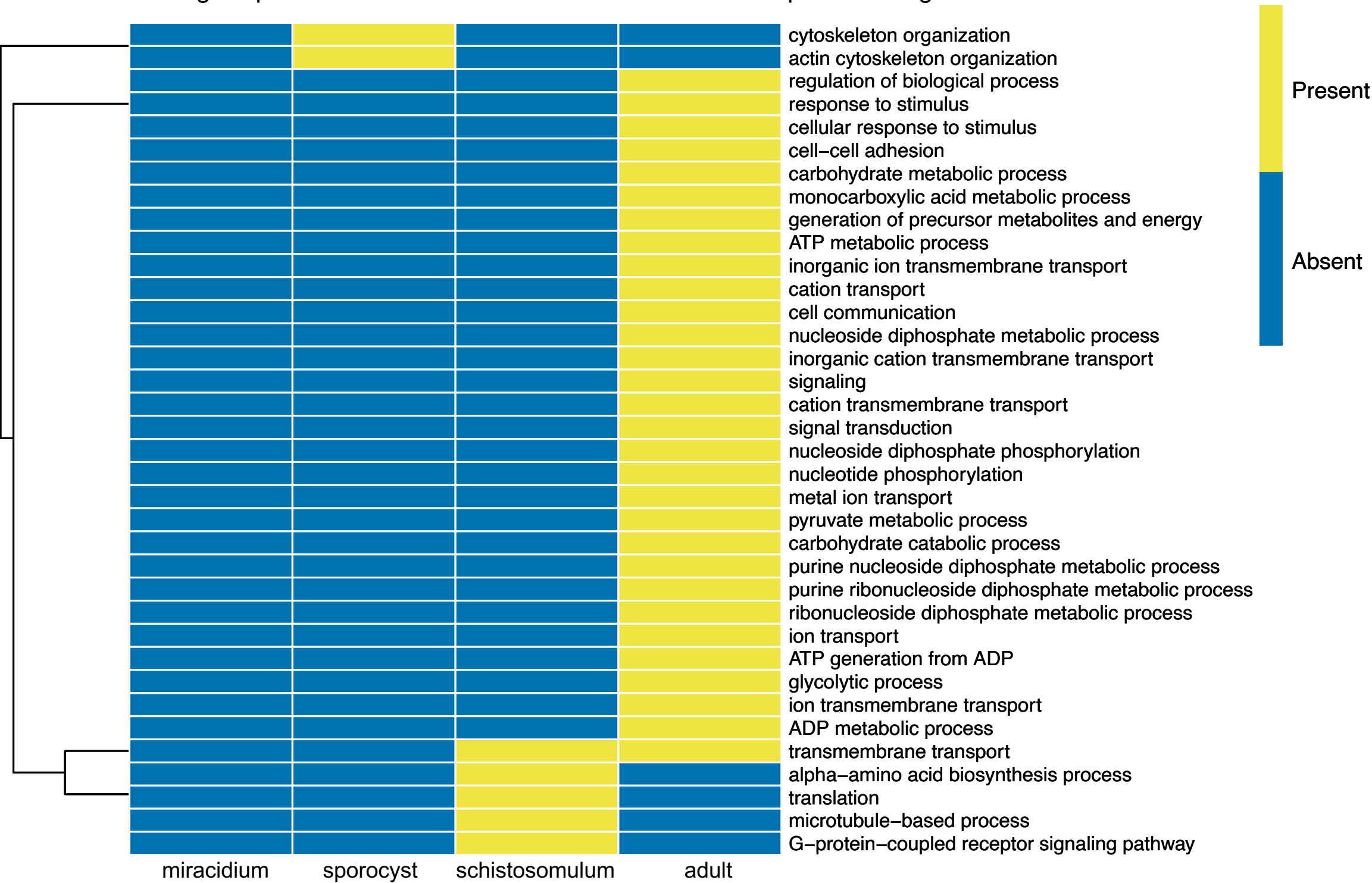

Supplement: Supplementary file 12 — Supplementary Information 12. [file 41598_2024_55790_MOESM12_ESM.pdf]

TOP 30 marker genes for Muscle cells in indicated *S. mansoni* developmental stages

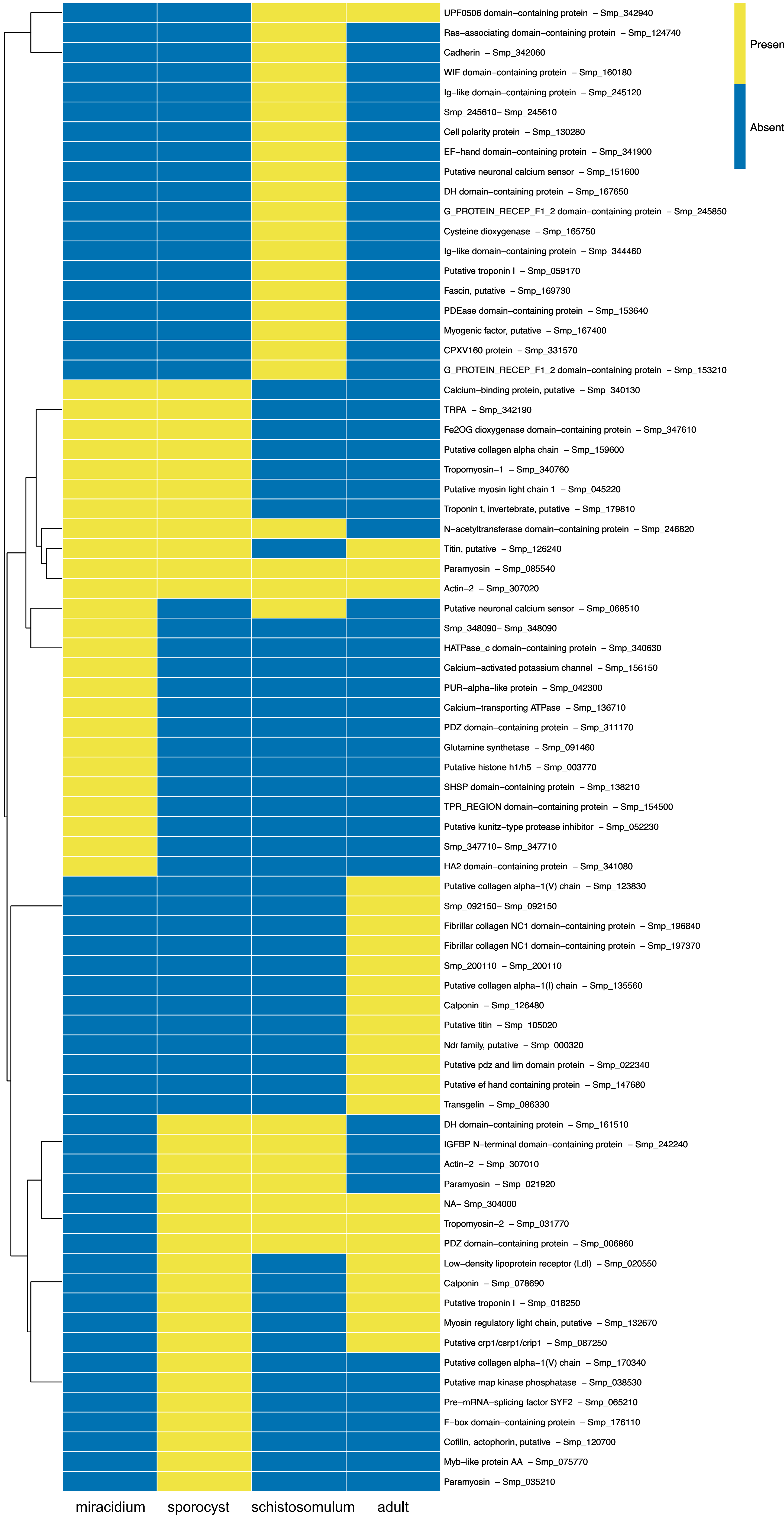

Supplement: Supplementary file 13 — Supplementary Information 13. [file 41598_2024_55790_MOESM13_ESM.pdf]

Biological processes in Neurons of *S. mansoni* developmental stages

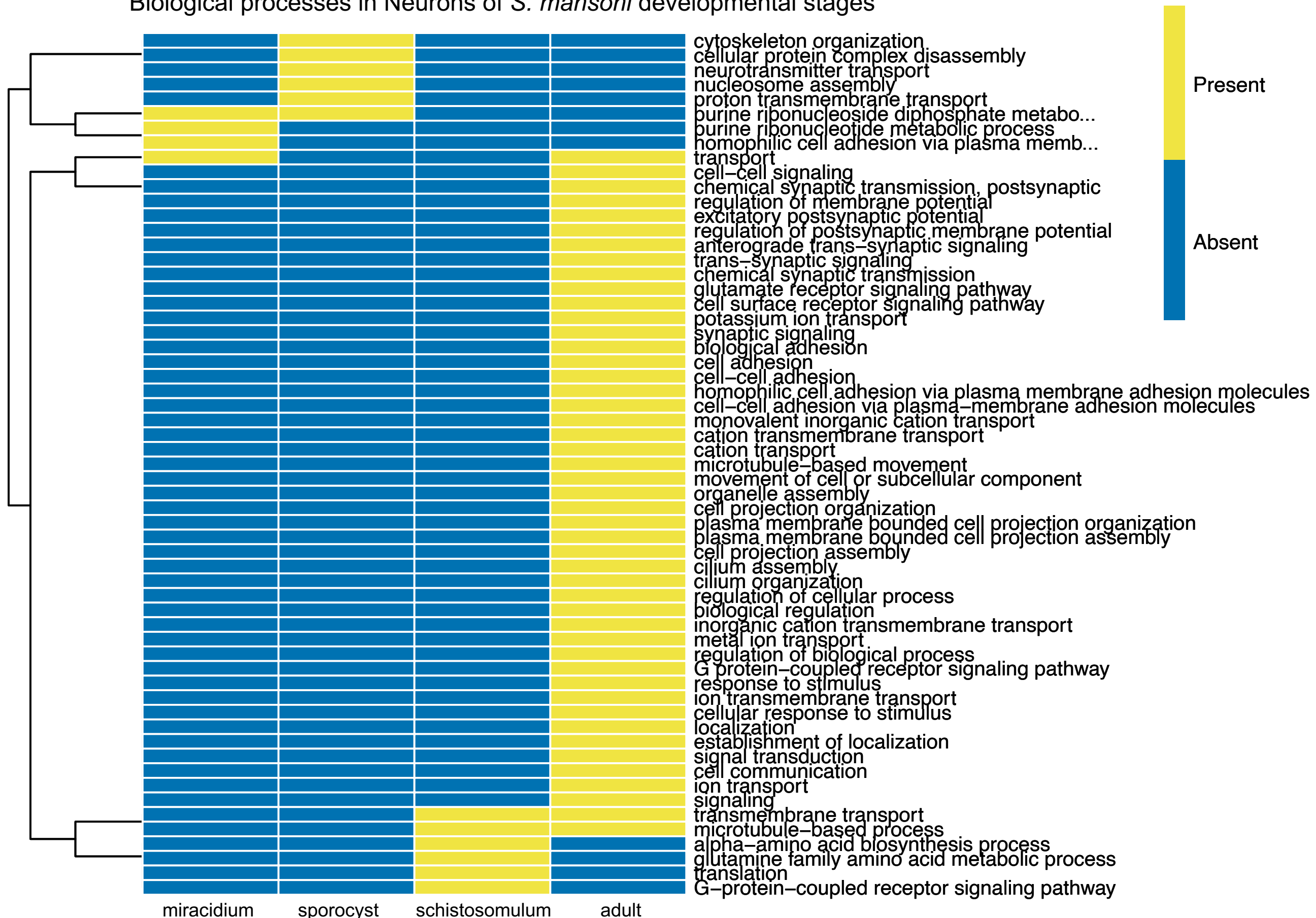

Supplement: Supplementary file 14 — Supplementary Information 14. [file 41598_2024_55790_MOESM14_ESM.pdf]

TOP 30 marker genes for Neurons in indicated *S. mansoni* developmental stages.

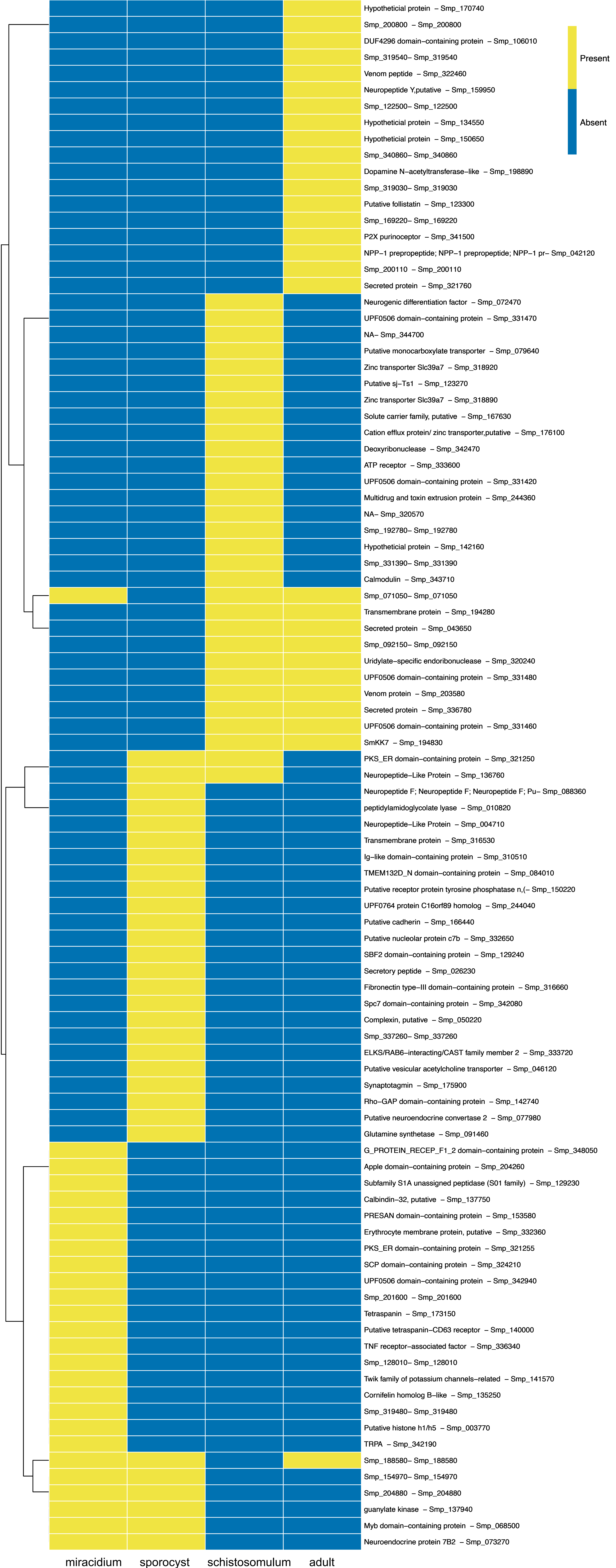

Supplement: Supplementary file 15 — Supplementary Information 15. [file 41598_2024_55790_MOESM15_ESM.pdf]

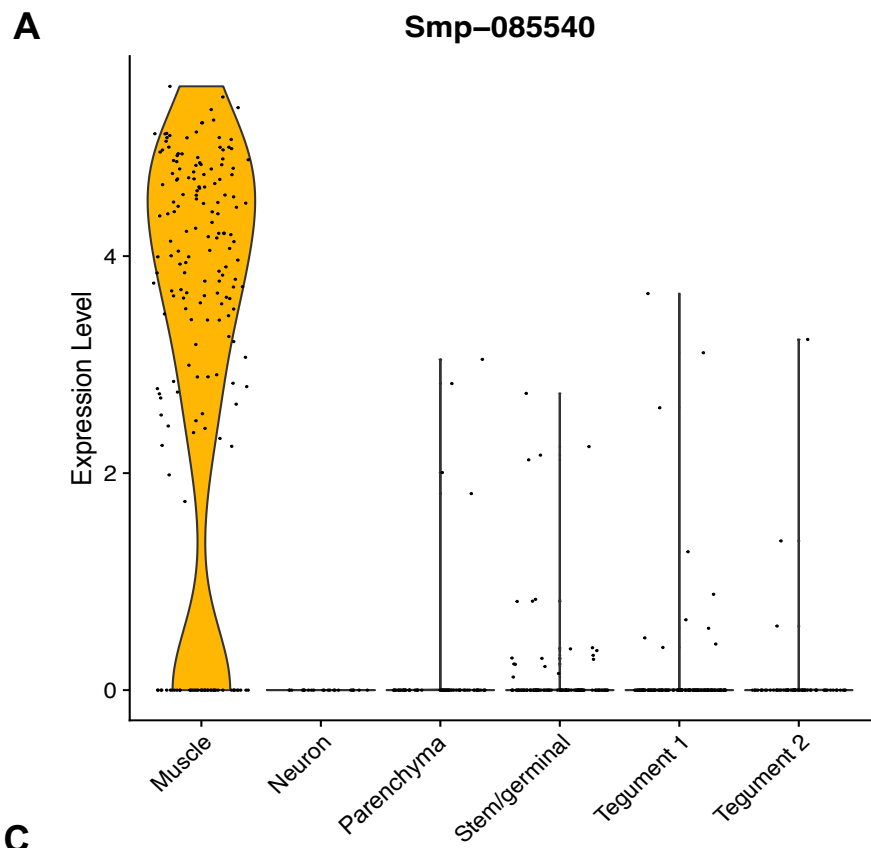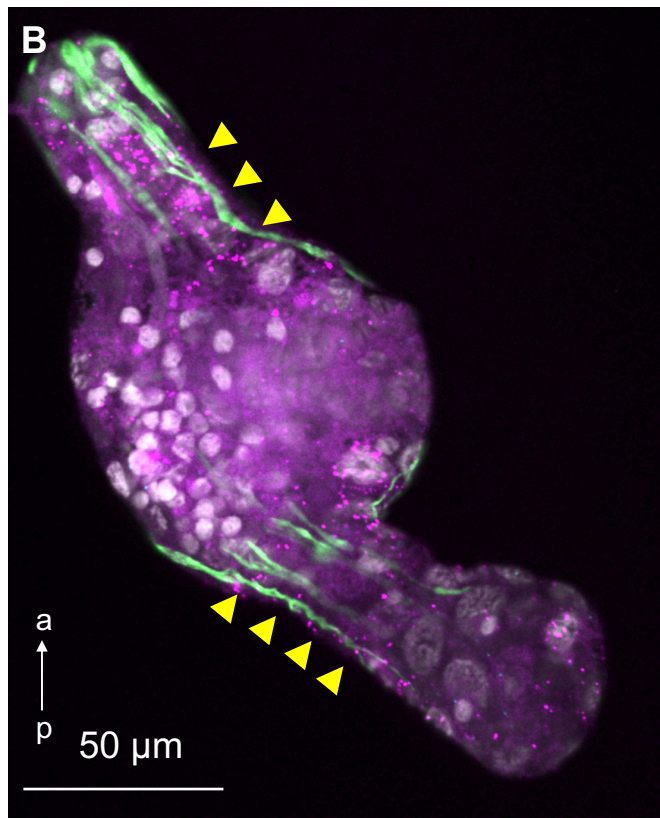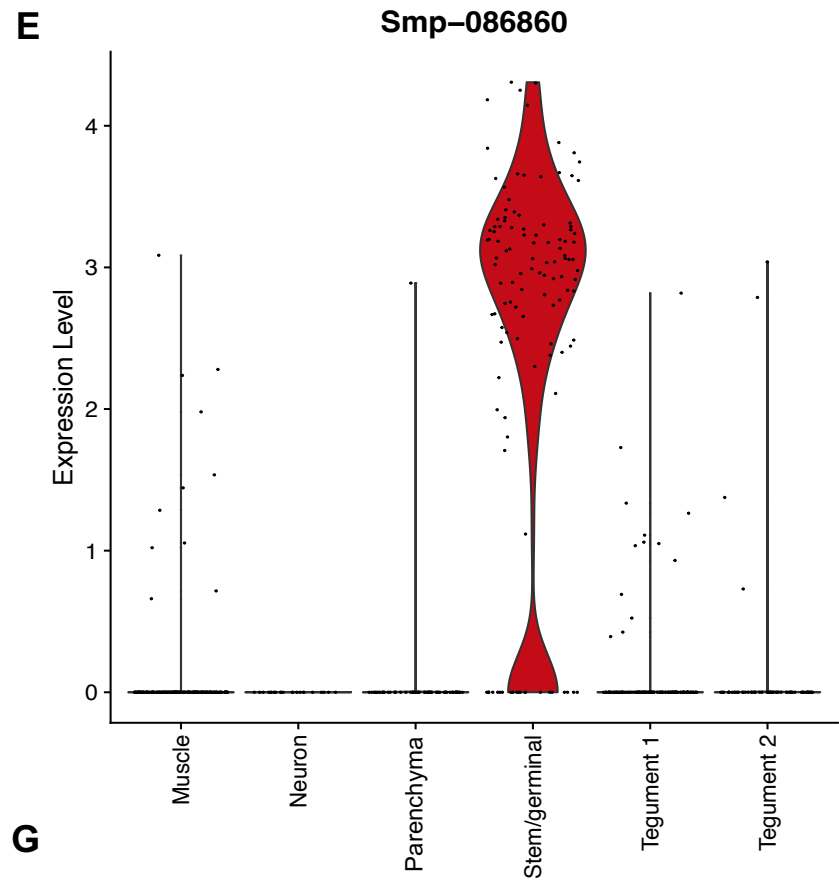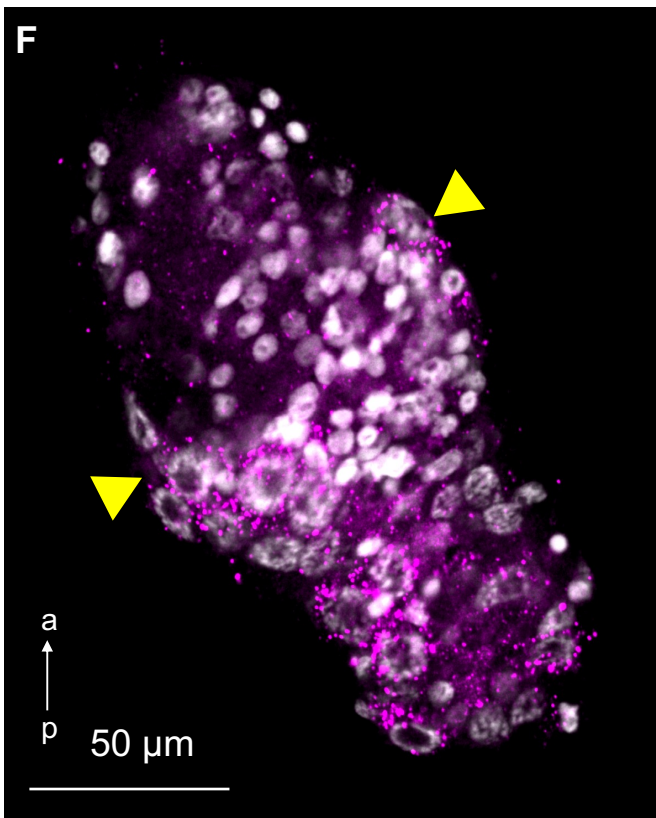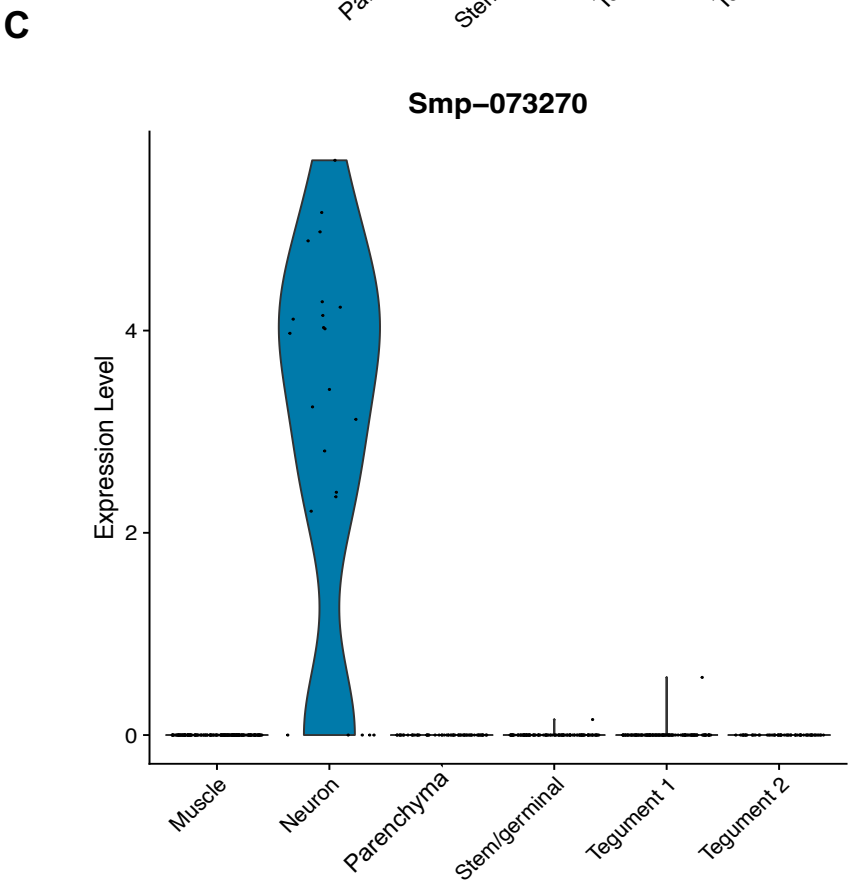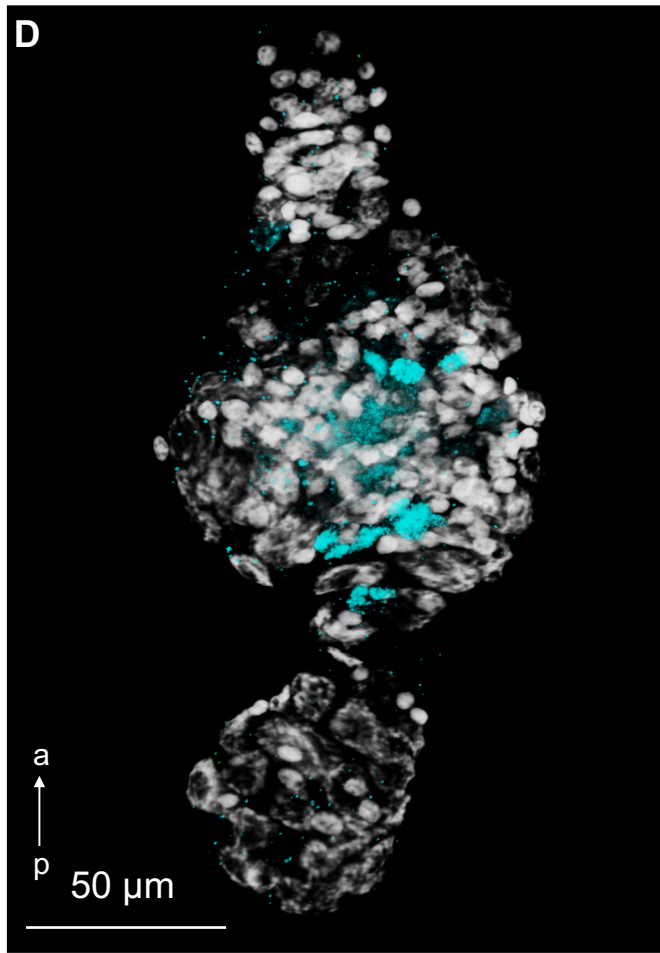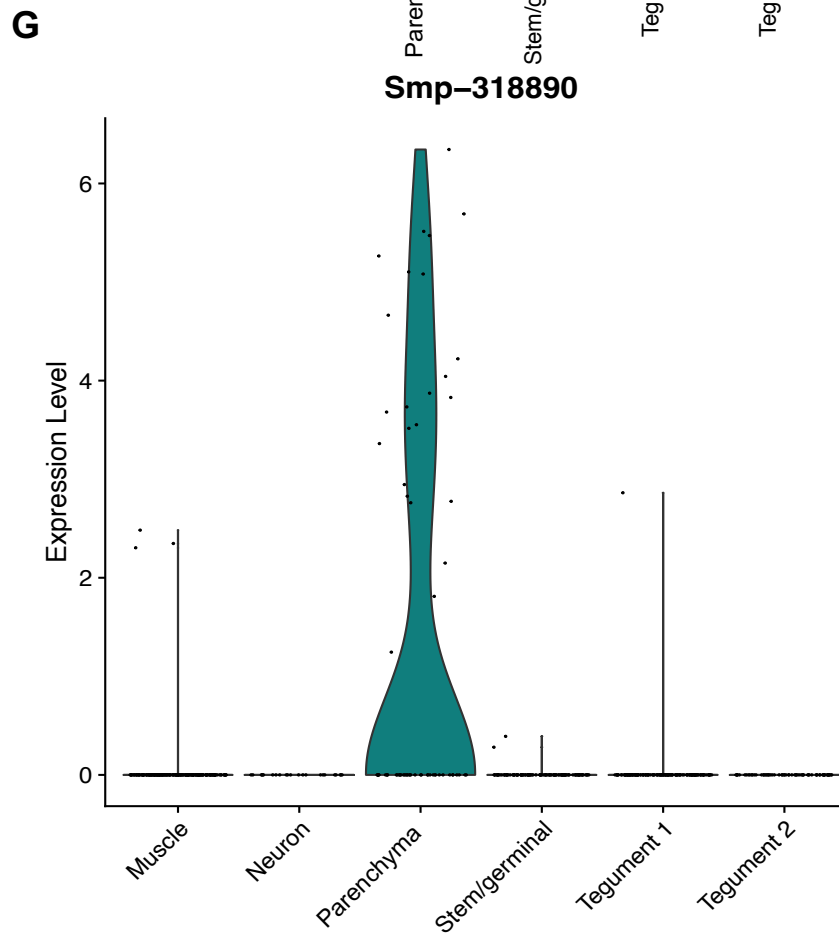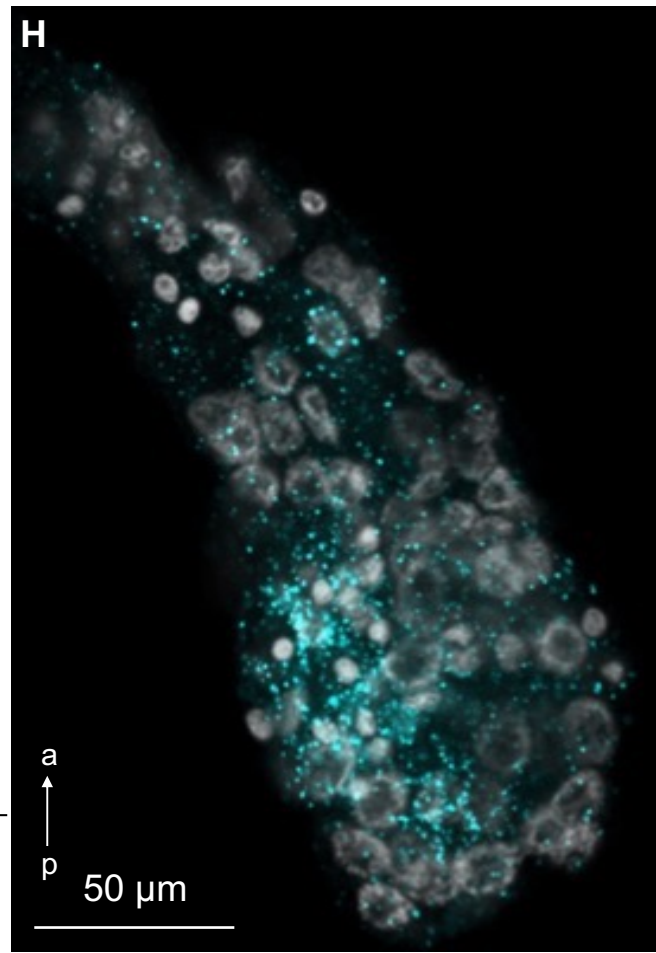

Supplement: Supplementary file 16 — Supplementary Information 16. [file 41598_2024_55790_MOESM16_ESM.pdf]

**A**

**Smp-163710**

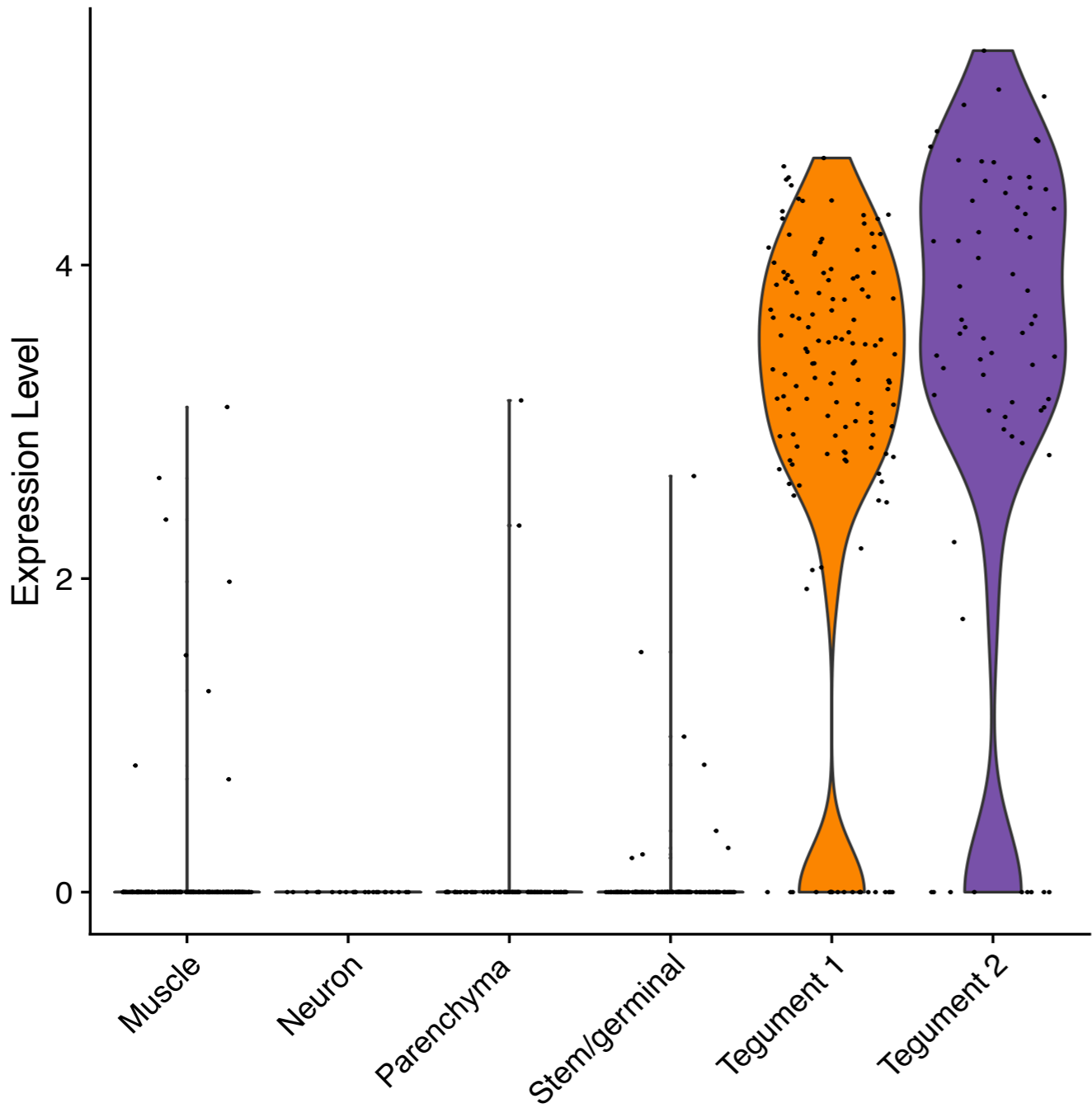

**B**

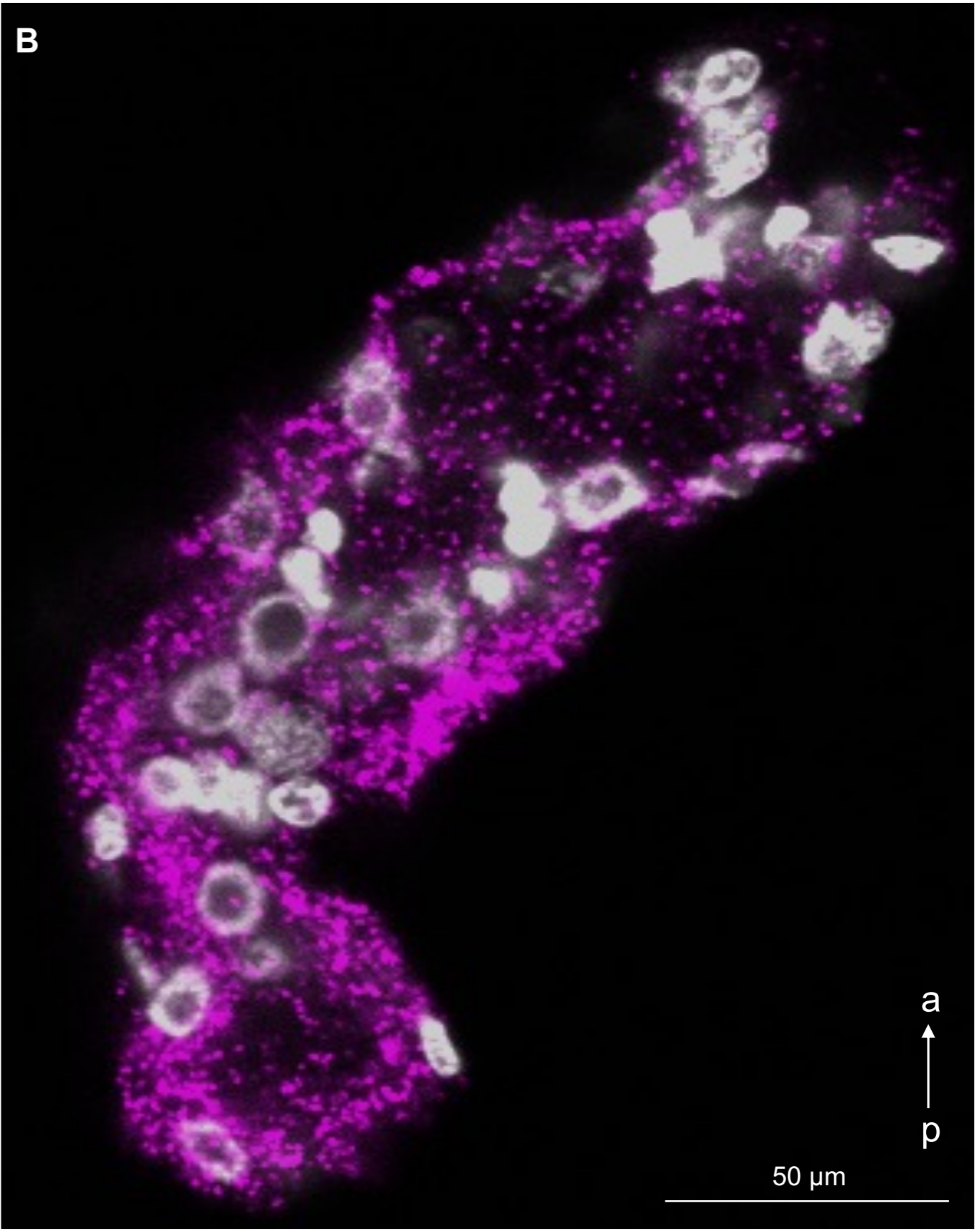

**C**

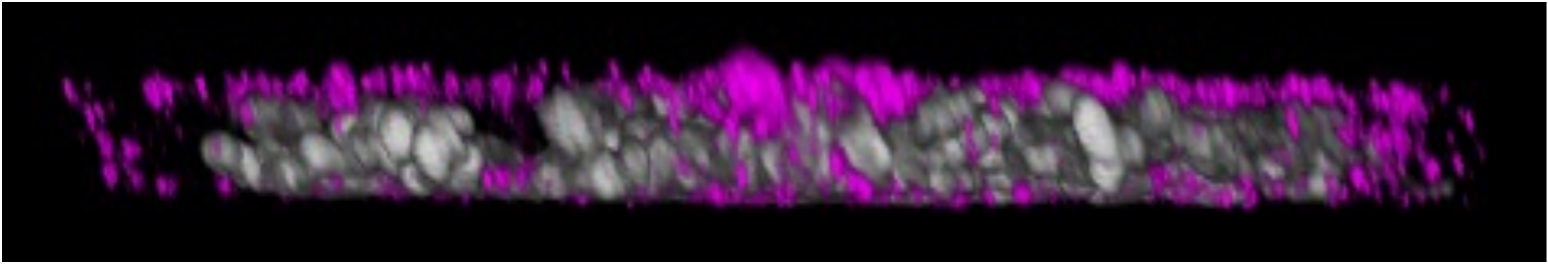

Supplement: Supplementary file 17 — Supplementary Information 17. [file 41598_2024_55790_MOESM17_ESM.pdf]

# *Kappa* stem cell sub-cluster markers

*ago2-1* (Smp\_179320)

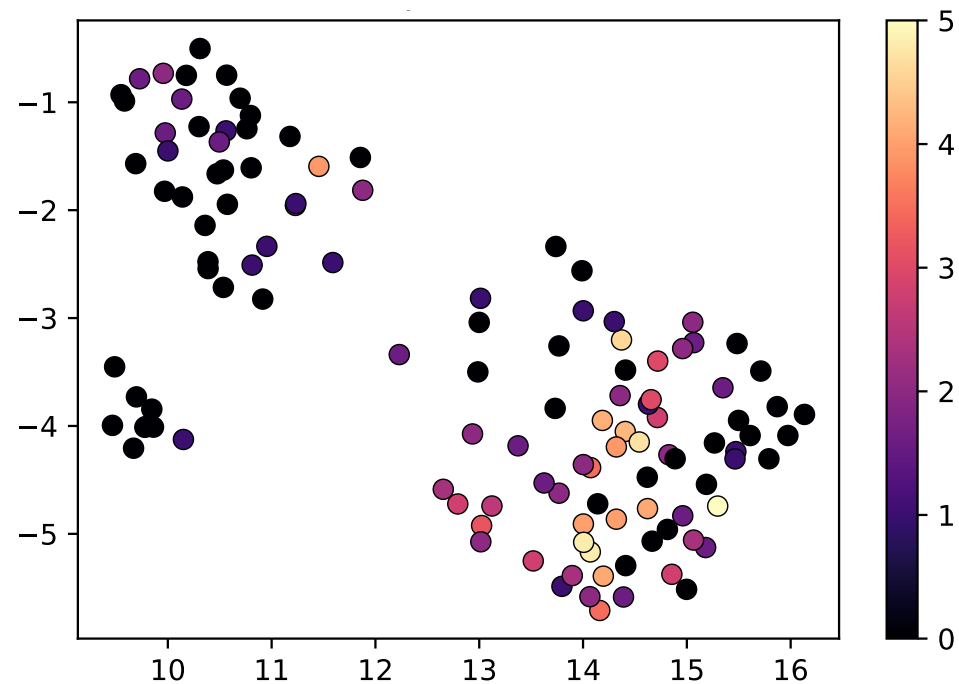

*nanos-2* (Smp\_051920)

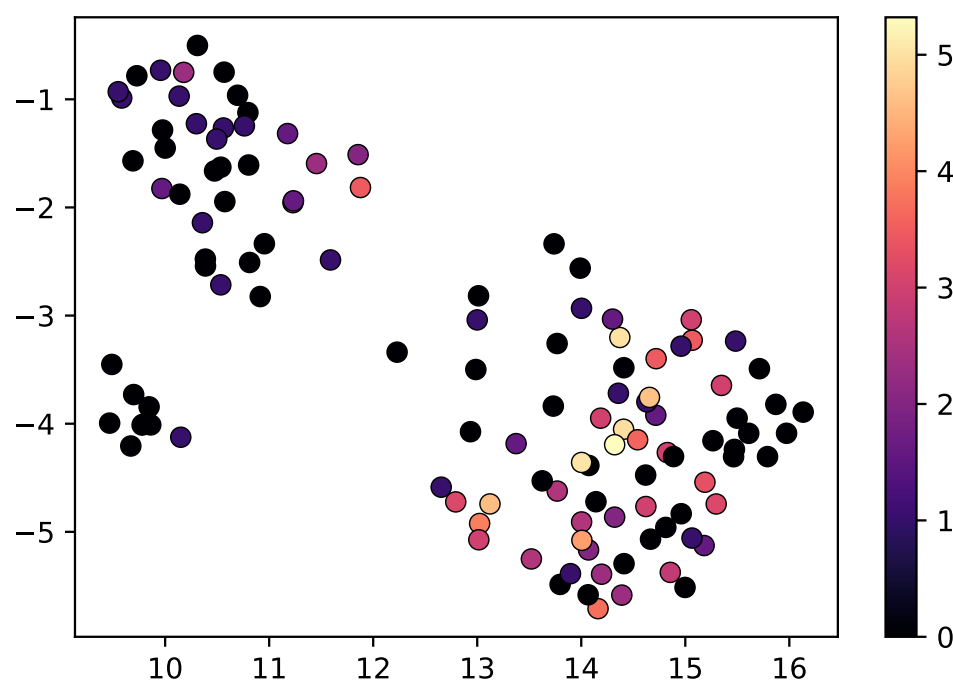

*klf* (Smp\_172480)

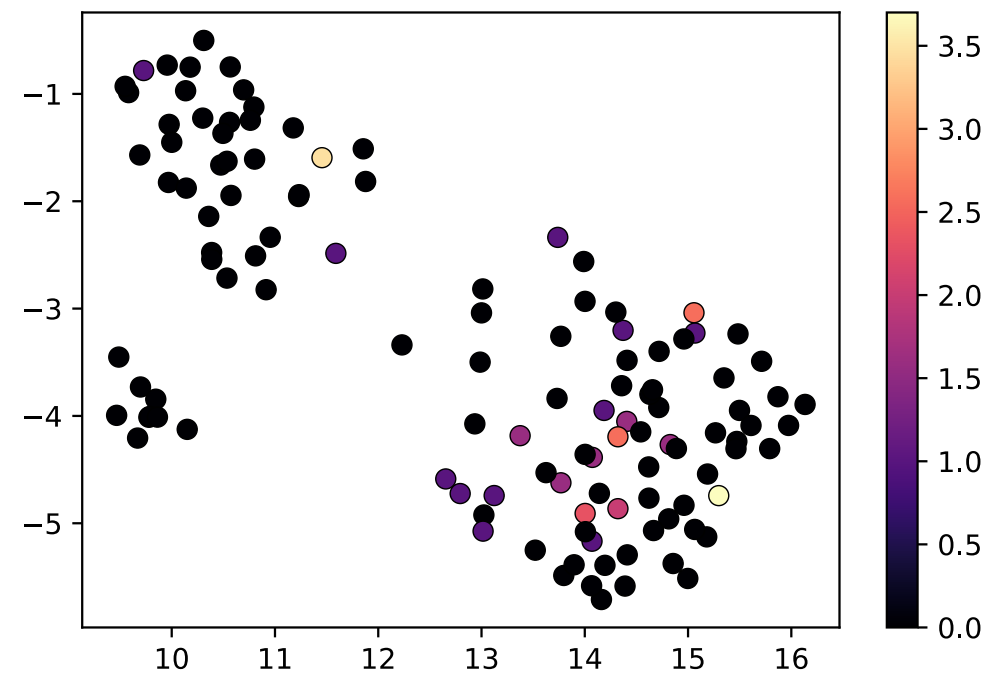

Supplement: Supplementary file 18 — Supplementary Information 18. [file 41598_2024_55790_MOESM18_ESM.pdf]

***Delta*** stem cell sub-cluster markers

*ago2-1* (Smp\_179320)

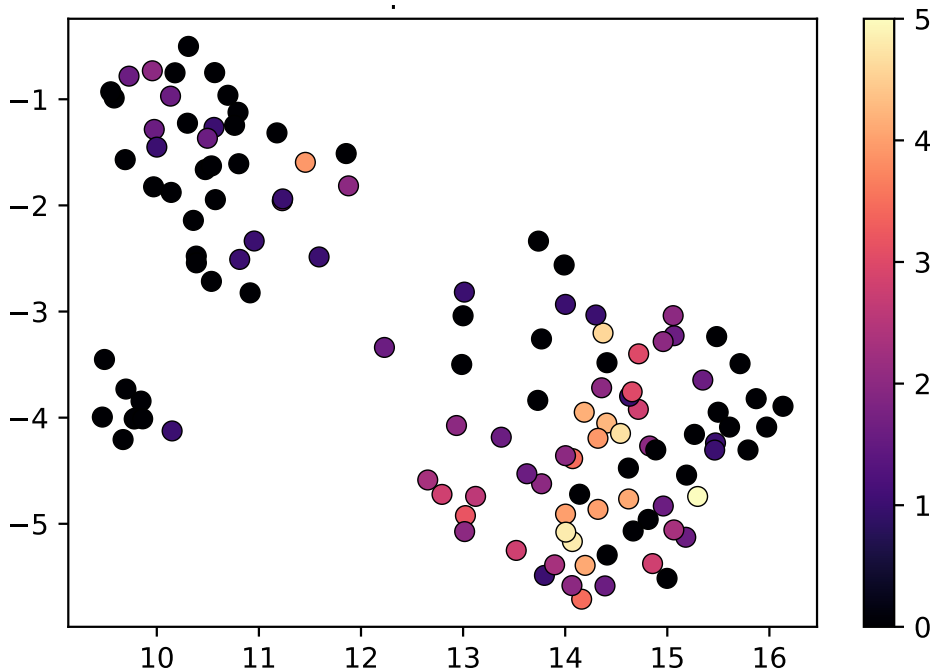

*nanos-2* (Smp\_051920)

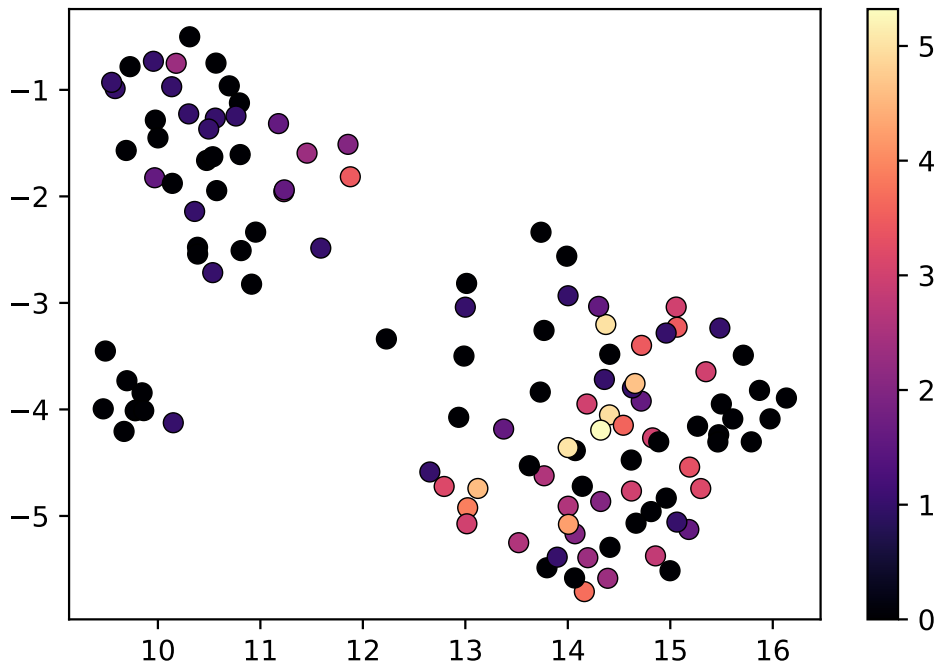

*fgfrA* (Smp\_175590)

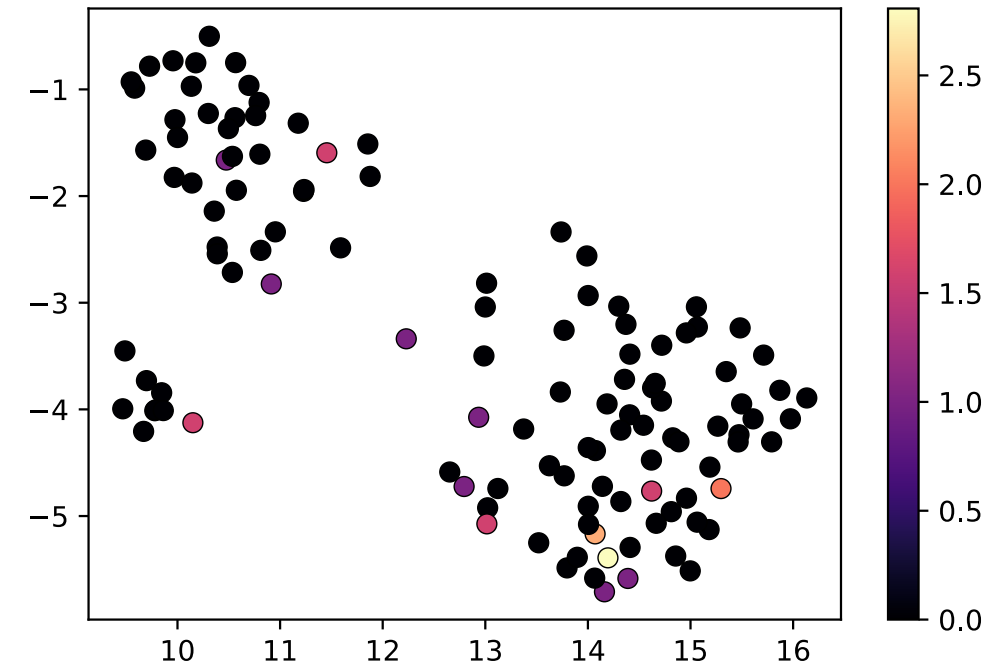

*p53* (Smp\_139530)

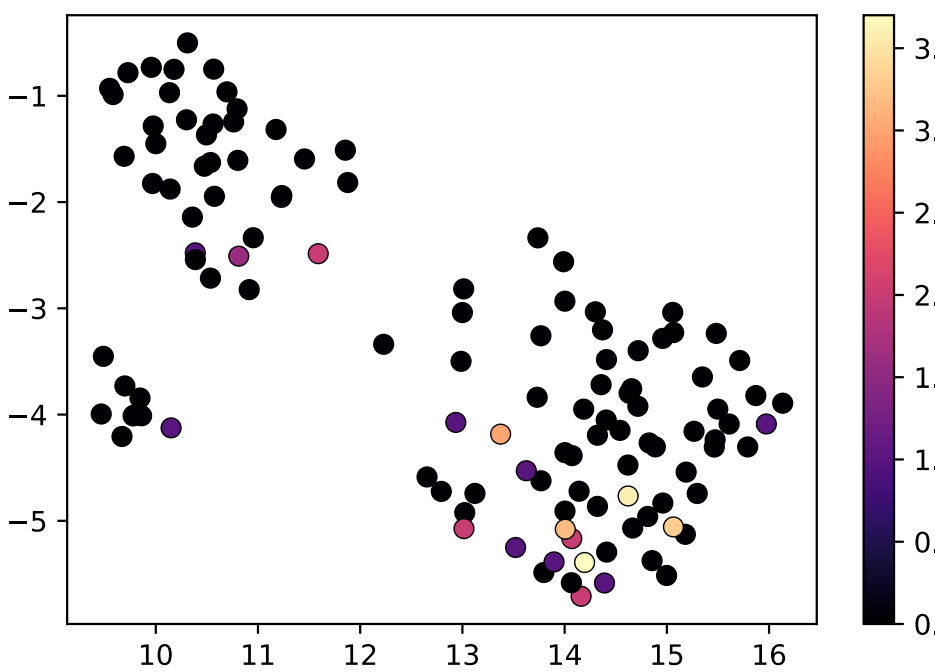

*fgfrB* (Smp\_157300)

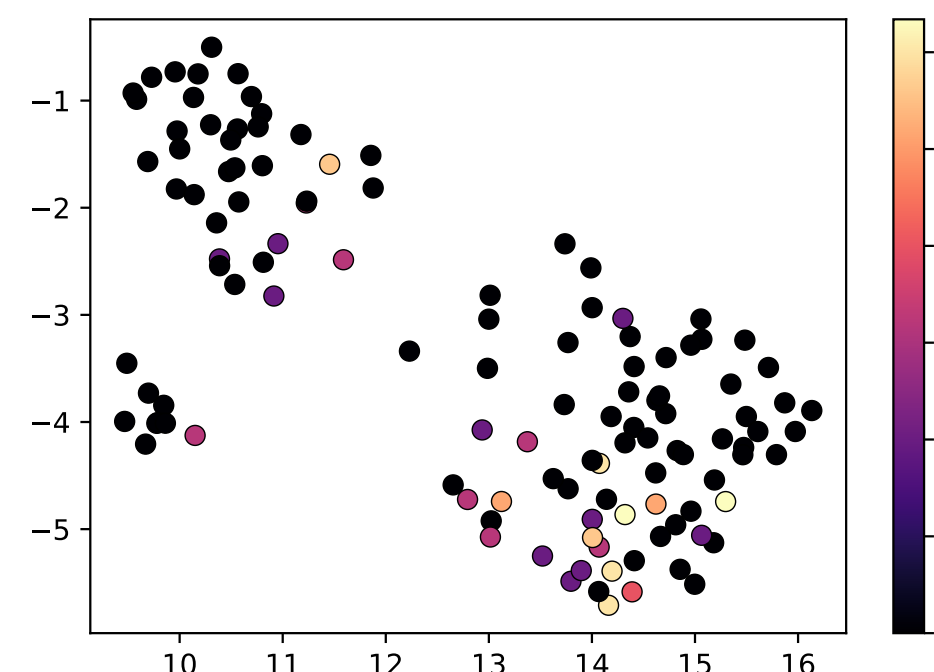

*Zfp-1* (Smp\_145470)

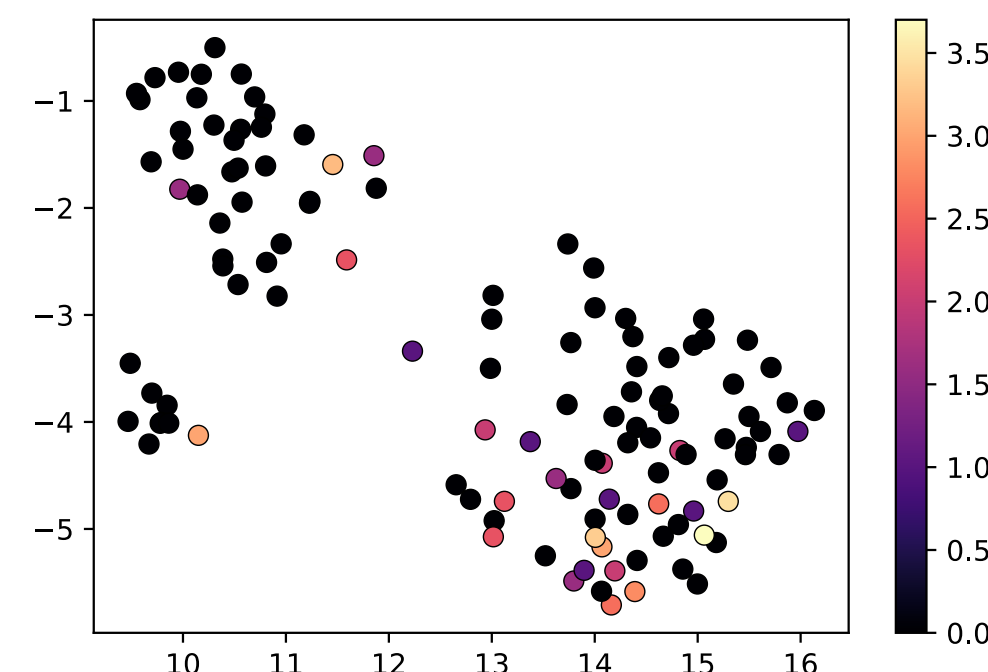

Supplement: Supplementary file 19 — Supplementary Information 19. [file 41598_2024_55790_MOESM19_ESM.pdf]

*Phi* stem cell sub-cluster markers

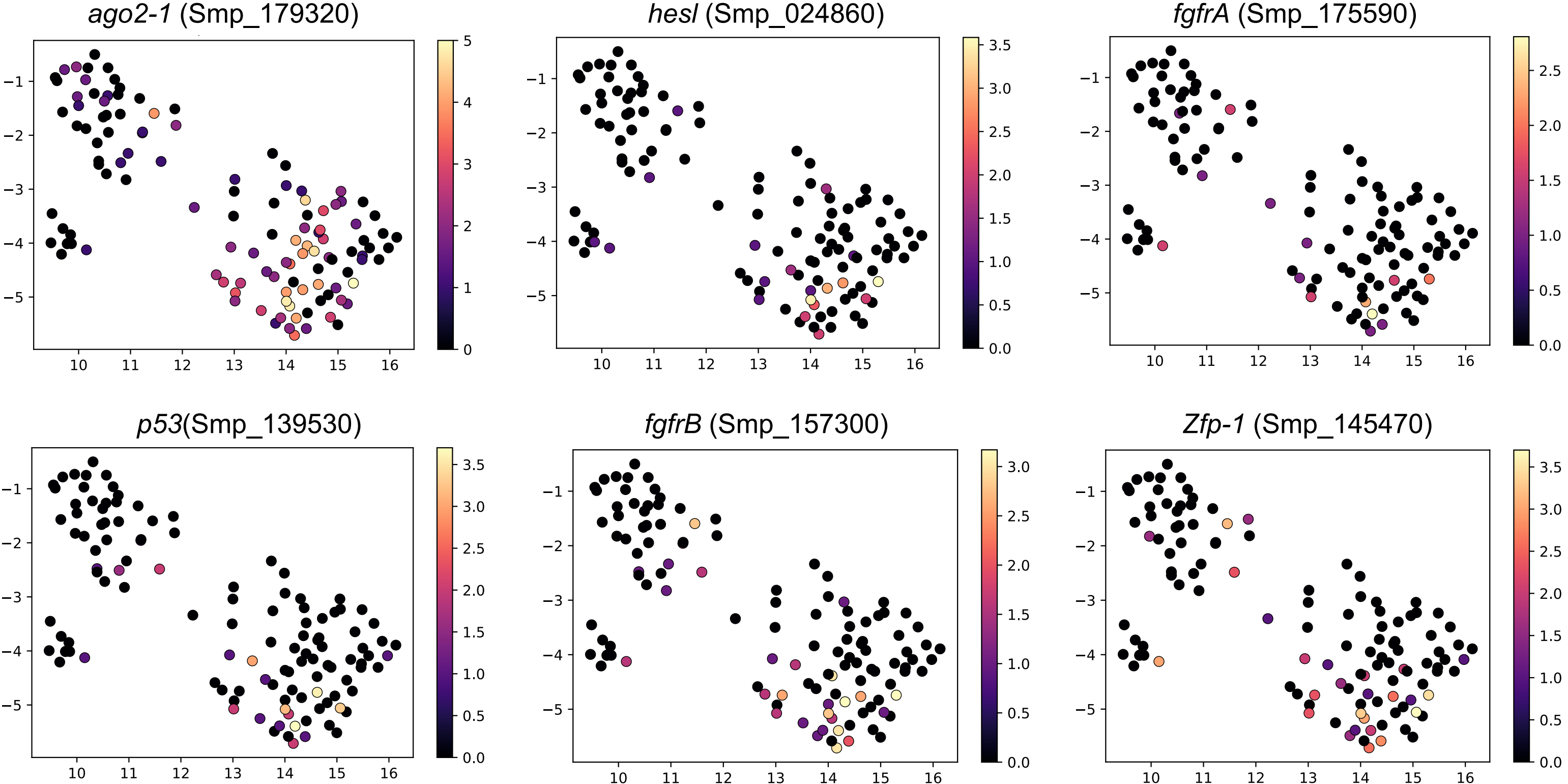

Supplement: Supplementary file 20 — Supplementary Information 20. [file 41598_2024_55790_MOESM20_ESM.pdf]

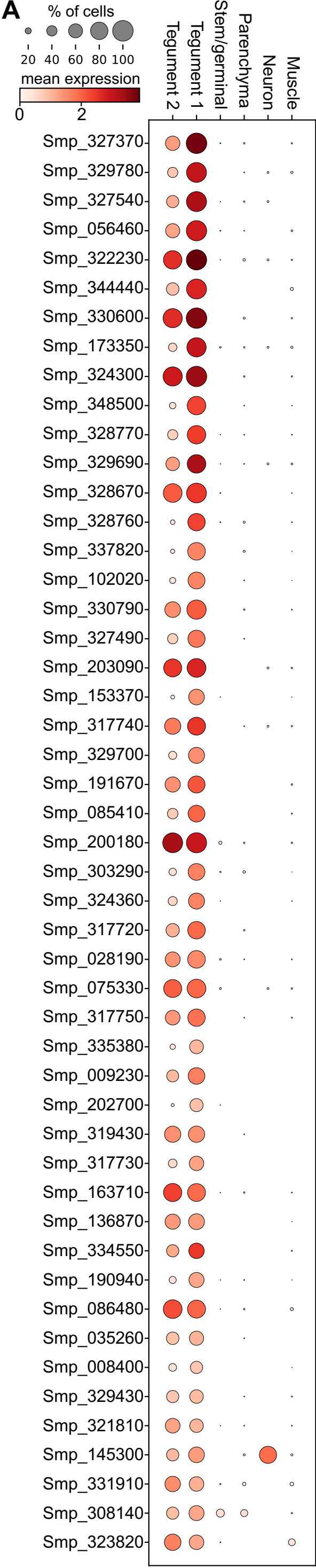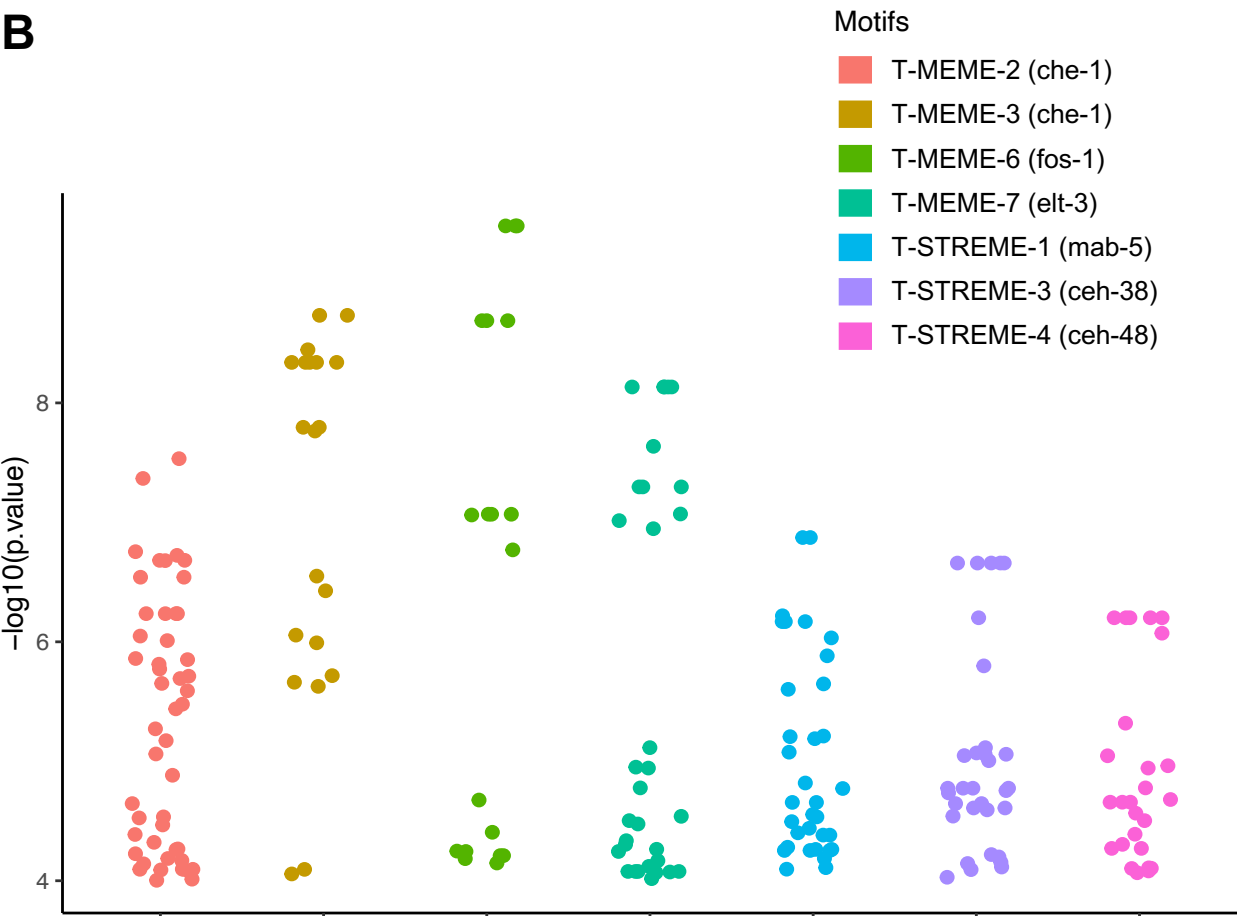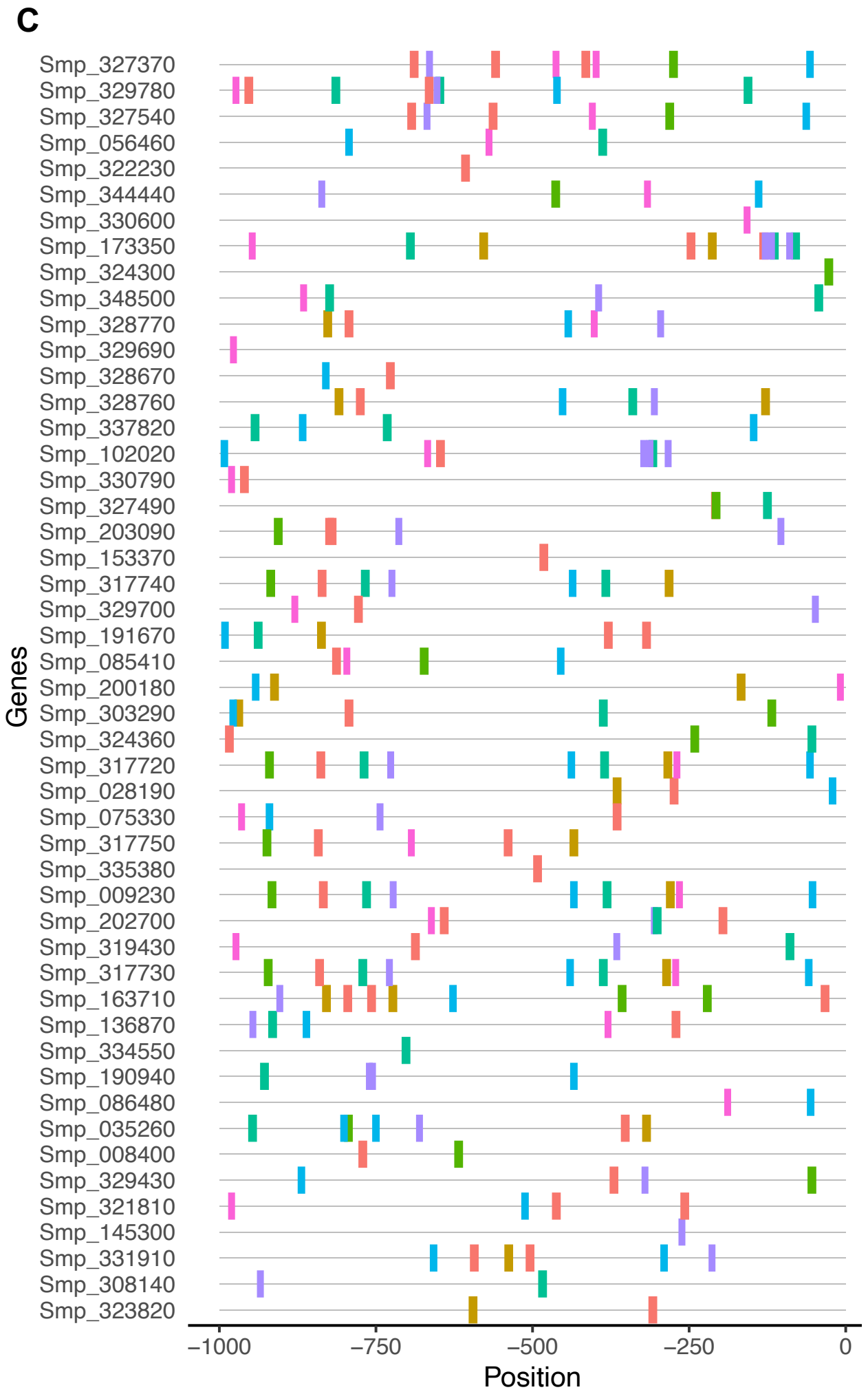

Supplement: Supplementary file 21 — Supplementary Information 21. [file 41598_2024_55790_MOESM21_ESM.pdf]

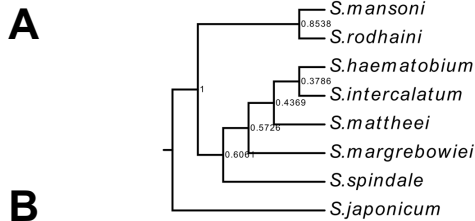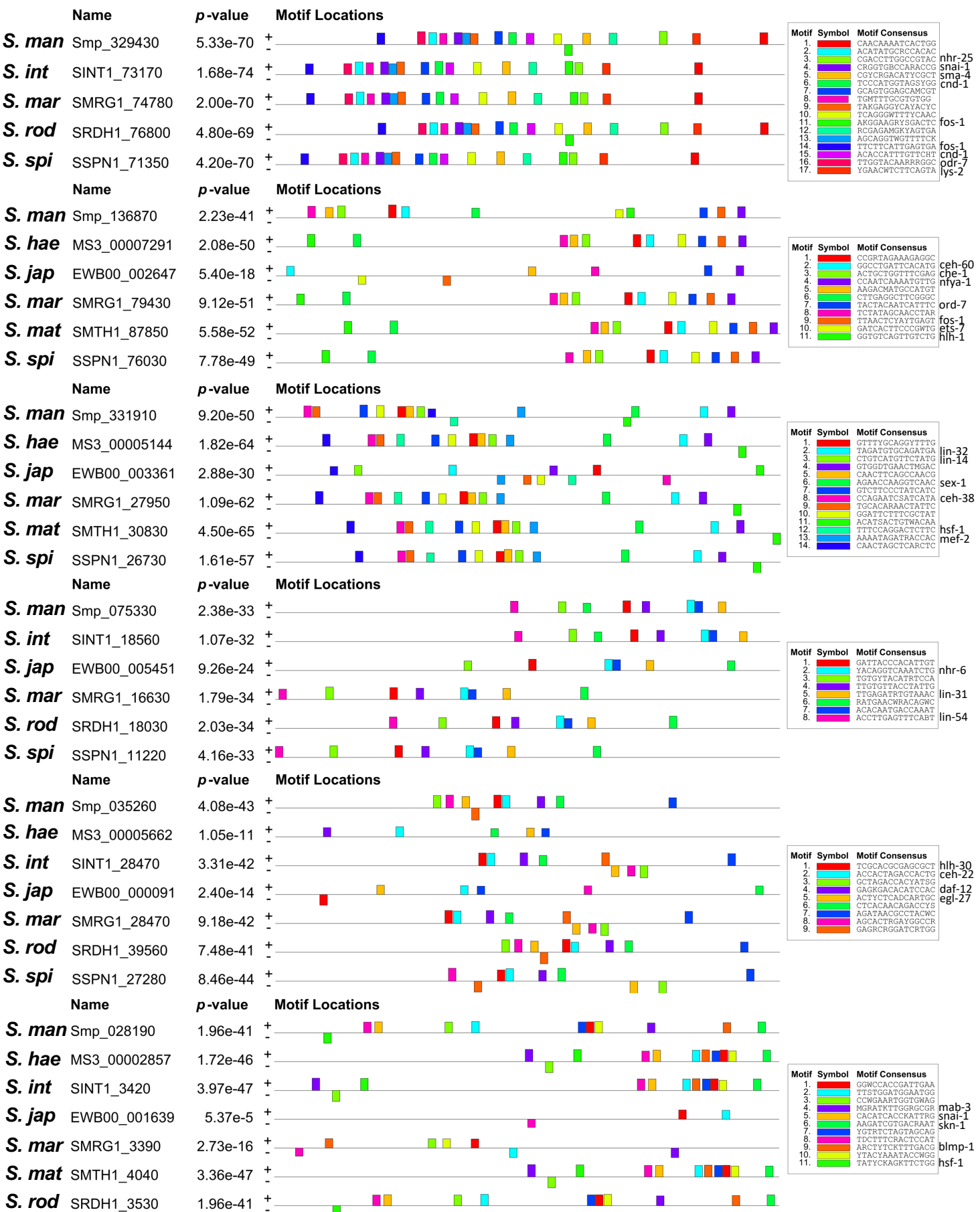

Supplement: Supplementary file 22 — Supplementary Information 22. [file 41598_2024_55790_MOESM22_ESM.pdf]

**A**

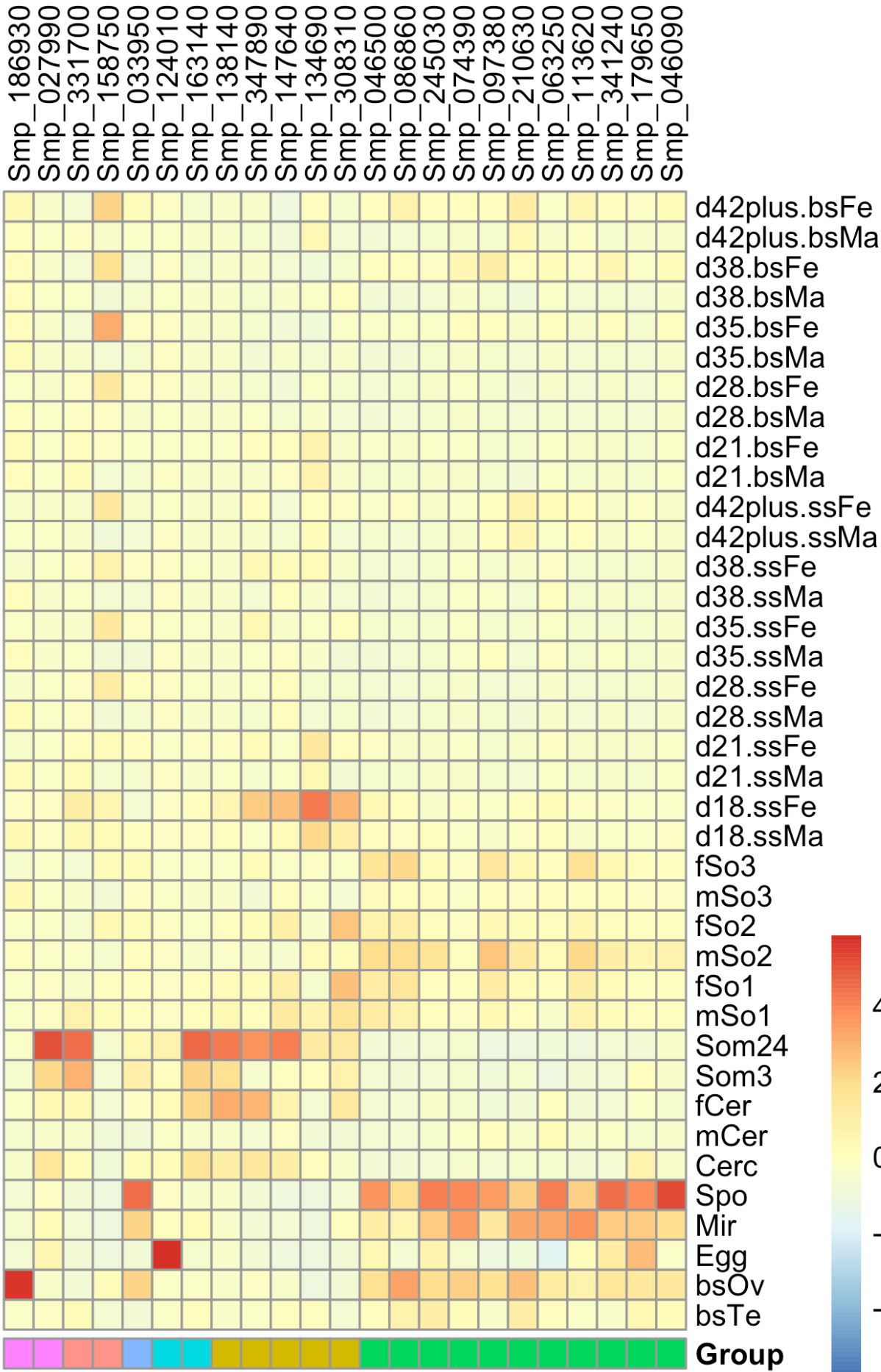

**B**

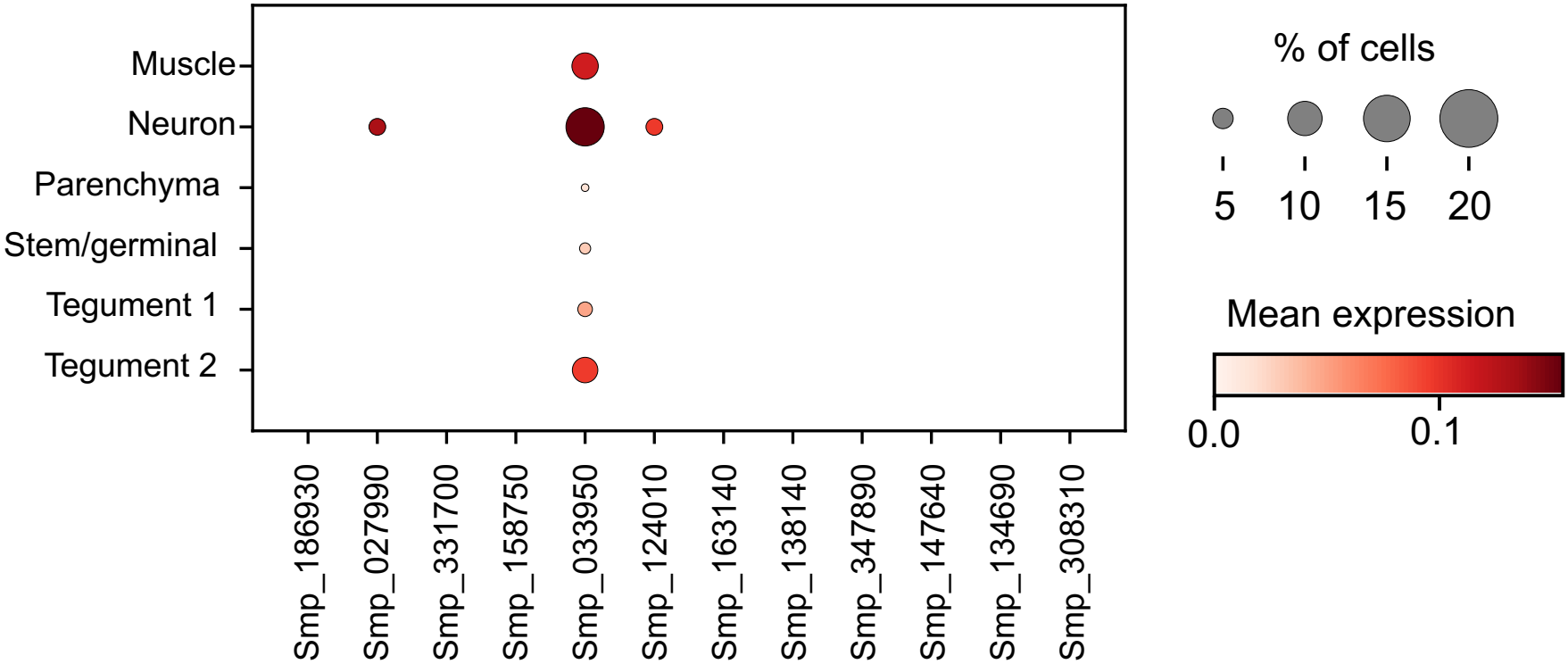

**C**

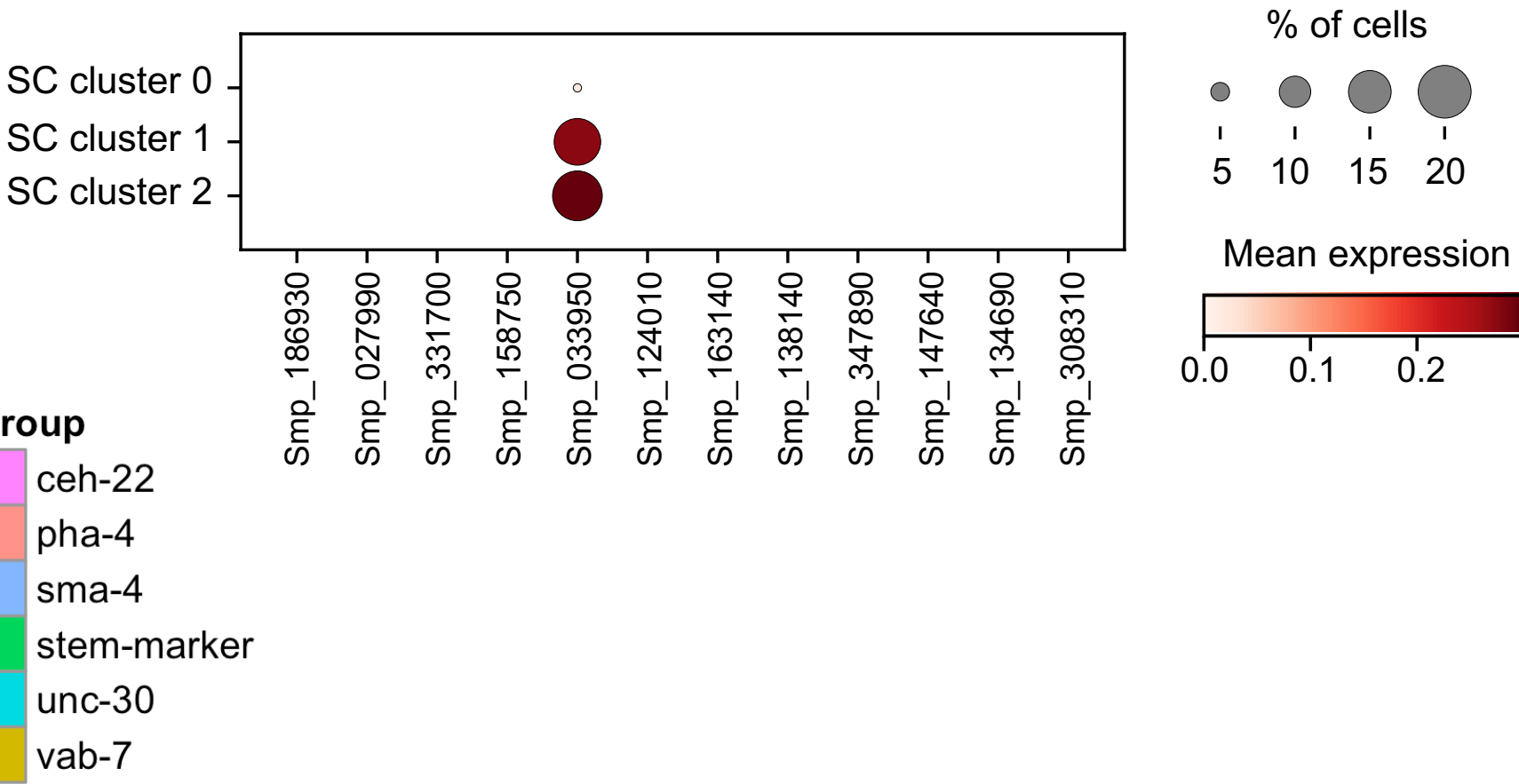

Supplement: Supplementary file 23 — Supplementary Information 23. [file 41598_2024_55790_MOESM23_ESM.pdf]

# *zfp-1* (Smp\_145570)

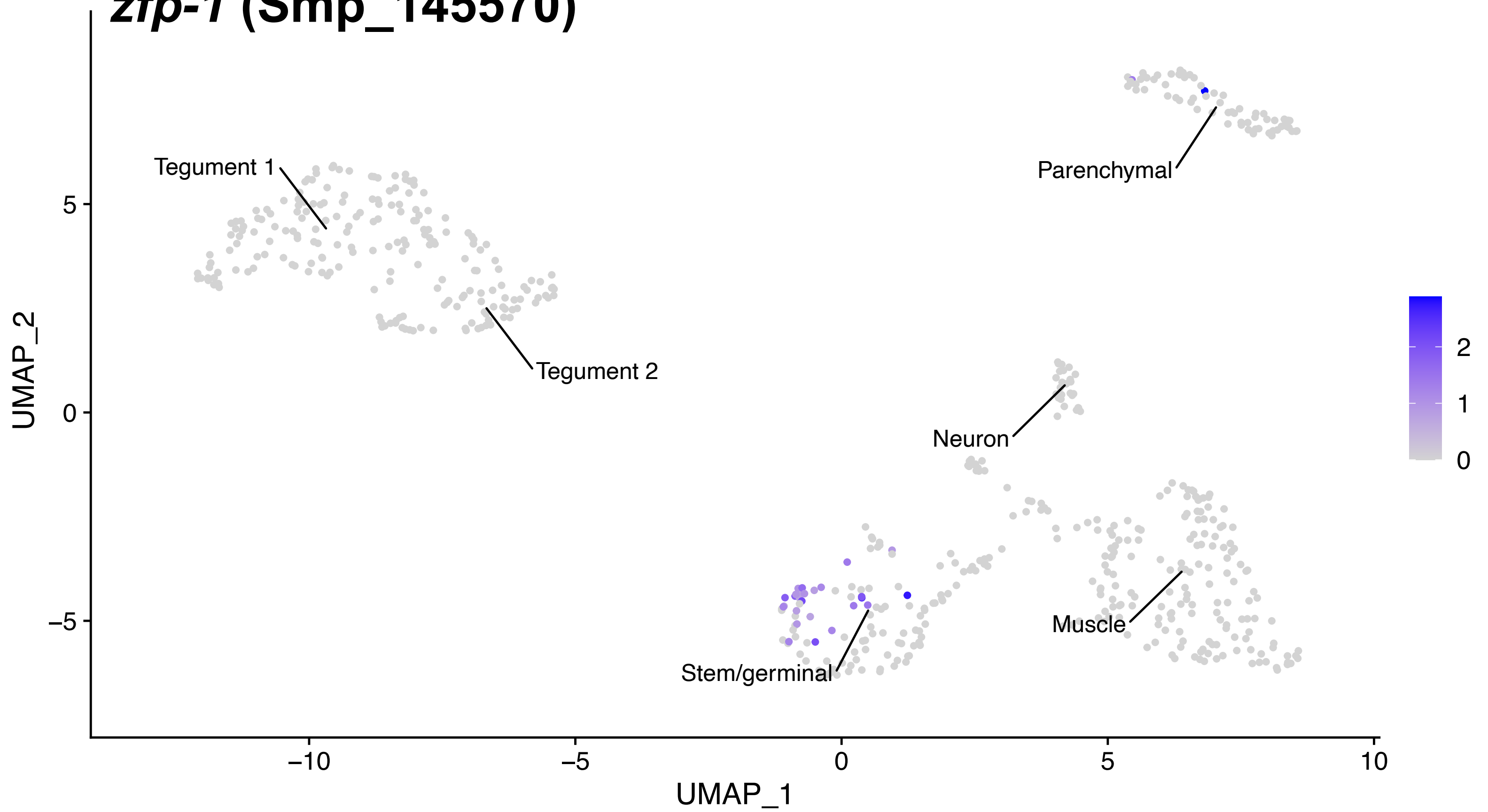

# *zfp-1-1* (Smp\_049580)

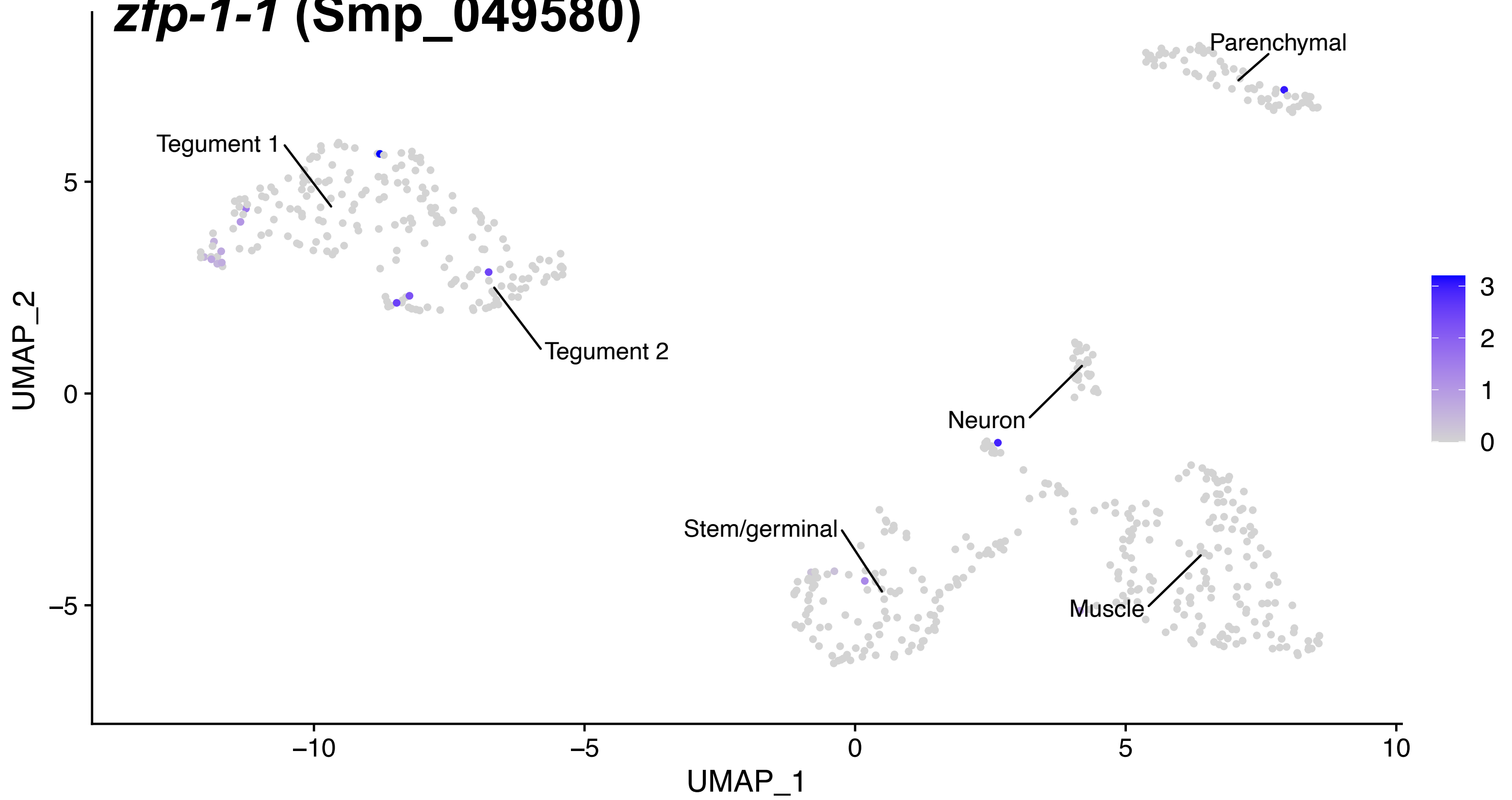

Supplement: Supplementary file 24 — Supplementary Information 24. [file 41598_2024_55790_MOESM24_ESM.pdf]
