# Supplementary material for: Single-cell transcriptomics of the human parasite Schistosoma mansoni first intra-molluscan stage reveals tentative tegumental and stem-cell regulators
Source: Sci Rep. 2024 Mar 12;14:5974. doi: 10.1038/s41598-024-55790-3 (PMC10933418; doi:10.1038/s41598-024-55790-3)
Supplement: Supplementary file 27 — Supplementary Information 27. [file 41598_2024_55790_MOESM27_ESM.docx]

**Supplementary Information**

**Supplementary Figures**

**Supplementary Figure S1. Live D5 mother sporocysts before processing for single cell RNA-seq or FISH.** Representative pictures of live *S. mansoni* mother sporocysts transformed from freshly hatched miracidia and cultured for 5 days in sporocyst medium under hypoxia at 28 ºC for 5 days. More than 95% of the parasites have fully transformed into mother sporocysts, and only a minority of parasites showed 1-3 cilia plates still attached to either the anterior or posterior pole (orange arrowheads). Scale bar: 100μm.

**Supplementary Figure S2. Nuclear segmentation of D5 mother sporocysts.** Nuclear segmentation of a representative D5 sporocyst used to generate the training data samples: pre-processed DAPI signal (**A**) and final segmentation map (**B**). **C.** Nuclear segmentation of 5 parasites revealed a total number of nuclei ranging from 112 to 254, with a mean of 168.6 and standard deviation of 57.68. Scale bar: 50μm.

**Supplementary Figure S3. Expression of serotonin receptors in muscle cells.** Uniform Manifold Approximation and Projection (UMAP) representation of 601 single cells from D5 sporocysts highlighting the expression of a previously characterised serotonin receptor (top panel - Sm5HTR) in intra-mammalian stages of *S. mansoni* ^23^. Bottom panel shows the expression of Smp_245850, *described as G_PROTEIN_RECEP_F1_2 domain-containing protein in WBPS. This gene resulted from merging Smp_156210 and Smp_148210 in the latest version of WBPS. Smp_148210 was previously described as a partial 5HT7-like sequence that lacked a transmembrane domain and was unlikely to be functional^23^.

**Supplementary Figure S4. Tegument 1 and Tegument 2 show different GO term enrichments. A.** TopGO summary of Biological Processes, Molecular Function, and Cellular Component terms differentially expressed genes (p_val_adj < 0.001) between teguments 1 (orange) and 2 (purple) subclusters. FDR: False Discovery Rate. **B.** Representatives of differentially expressed genes between tegument 1 and 2. Top panel: Smp_348500, upregulated and enriched in tegument 1 cells, described as Plexin domain-containing protein in WBPS. Bottom panel: Smp_121950, upregulated in tegument 2 cells, described as arrestin_C domain-containing protein in WBPS. The expression of tegument 2 upregulated genes is also evident in tegument 1 cells. Full data available in **Supplementary Tables S6-S8.**

**Supplementary Figure S5. TopGO summary of Biological Processes in the Tegument cluster(s) of *S. mansoni* developmental stages.** Clustered heatmap showing presence (yellow)/ absence (blue) of the tegument cluster(s) topGO terms in the miracidia, sporocysts, schistosomula and adult worms, as indicated. Full data available in **Supplementary Table S9**.

**Supplementary Figure S6. TOP 30 marker genes for Tegument clusters in indicated *S. mansoni* developmental stages.** Clustered heatmap showing presence (yellow)/ absence (blue) of common top marker genes of tegument cluster(s) for sporocysts, miracidia, schistosomula, and adult stages. ‘Top’ was defined by log-fold change in miracidia, sporocysts, and adults, and average.diff in schistosomula (see Methods). Full data available in **Supplementary Table S10.**

**Supplementary Figure S7. TopGO summary of Biological Processes in the Stem/germinal cell cluster(s) of *S. mansoni* developmental stages.** Clustered heatmap showing presence (yellow)/ absence (blue) of the stem/germinal cell cluster(s) topGO terms in the miracidia, sporocysts, schistosomula and adult worms, as indicated. Full data available in **Supplementary Table S9**.

**Supplementary Figure S8.** **TOP 30 marker genes for Stem/germinal cell clusters in indicated *S. mansoni* developmental stages.** Clustered heatmap showing presence (yellow)/ absence (blue) of common top marker genes of stem/germinal cell cluster(s) for sporocysts, miracidia, schistosomula, and adult stages. ‘Top’ was defined by log-fold change in miracidia, sporocysts, and adults, and average.diff in schistosomula (see Methods). Full data available in **Supplementary Table S11.**

**Supplementary Figure S9. TopGO summary of Biological Processes in the Parenchyma cluster(s) of *S. mansoni* developmental stages.** Clustered heatmap showing presence (yellow)/ absence (blue) of the parenchyma cell cluster(s) topGO terms in the miracidia, sporocysts, schistosomula and adult worms, as indicated. Full data available in **Supplementary Table S9**.

**Supplementary Figure S10.** **TOP 30 marker genes for Parenchyma clusters in indicated *S. mansoni* developmental stages.** Clustered heatmap showing presence (yellow)/ absence (blue) of common top marker genes of parenchyma cell cluster(s) for sporocysts, miracidia, schistosomula, and adult stages. ‘Top’ was defined by log-fold change in miracidia, sporocysts, and adults, and average.diff in schistosomula (see Methods). Full data available in **Supplementary Table S12.**

**Supplementary Figure S11. TopGO summary of Biological Processes in the Muscle cluster(s) of *S. mansoni* developmental stages.** Clustered heatmap showing presence (yellow)/ absence (blue) of the muscle cell cluster(s) topGO terms in the miracidia, sporocysts, schistosomula and adult worms, as indicated. Full data available in **Supplementary Table S9**.

**Supplementary Figure S12.** **TOP 30 marker genes for Muscle clusters in indicated *S. mansoni* developmental stages.** Clustered heatmap showing presence (yellow)/ absence (blue) of common top marker genes of muscle cell cluster(s) for sporocysts, miracidia, schistosomula, and adult stages. ‘Top’ was defined by log-fold change in miracidia, sporocysts, and adults, and average.diff in schistosomula (see Methods). Full data available in **Supplementary Table S13.**

**Supplementary Figure S13. TopGO summary of Biological Processes in the Neuron cluster(s) of *S. mansoni* developmental stages.** Clustered heatmap showing presence (yellow)/ absence (blue) of the neuron cell cluster(s) topGO terms in the miracidia, sporocysts, schistosomula and adult worms, as indicated. Full data available in **Supplementary Table S9**.

**Supplementary Figure S14.** **TOP 30 marker genes for neuron clusters in indicated *S. mansoni* developmental stages.** Clustered heatmap showing presence (yellow)/ absence (blue) of common top marker genes of neuron cell cluster(s) for sporocysts, miracidia, schistosomula, and adult stages. ‘Top’ was defined by log-fold change in miracidia, sporocysts, and adults, and average.diff in schistosomula (see Methods). Full data available in **Supplementary Table S14.**

**Supplementary Figure S15. Spatial validation of cell clusters. A.** Violin plot showing the expression level of the muscle marker *myosin heavy chain* (Smp_085540) across the six cell clusters. **B.** Muscle cells expressing *myosin heavy chain* (*S*mp_085540; magenta) revealed by FISH, co-localised with phalloidin-stained actin filaments (green). Yellow arrowheads indicate co-localisation of *myosin heavy chain* and actin filaments. **C.** Violin plot showing the expression level of the neuron marker *neuroendocrine protein 7b2* (Smp_073270) across the six cell clusters. **D.** Neuron cluster cells expressing neuroendocrine *protein 7b2* (Smp_073270; cyan) revealed by FISH (same specimen as the one shown in Figure 2B). **E.** Violin plot showing the expression level of the stem/germinal marker *histone H2A* (Smp_086860) across the six cell clusters. **F.** Stem/germinal cells expressing *histone H2A* (Smp_086860; magenta) revealed by FISH Yellow arrowheads indicate representative Stem/germinal cells located in the medial region towards the surface of the animal. **G.** Violin plot showing the expression level of the parenchymal marker *hypothetical protein* (Smp_318890) across the six cell clusters. **H.** Parenchyma cells expressing *hypothetical protein* (*Smp_318890*; cyan) revealed by FISH. DAPI staining in grey. Scale bar: 50μm, a←p: anterior-posterior axis (Panels B, D, F, H)

**Supplementary Figure S16. Spatial validation of the tegument clusters.** **A.** Violin plot showing the expression level of the pan-tegumental micro-exon gene 6 or *MEG-6* (Smp_163710) across the 6 cell clusters. **B.** FISH of micro-exon gene 6 or *MEG-6* (Smp_163710)- expressing cells (magenta) identified the tegument clusters. DAPI staining in grey. Scale bar: 50μm, a←p: anterior-posterior axis. **C.** Sagittal projection of a parasite showing the tegumental expression of *MEG-6*. DAPI staining in grey.

**Supplementary Figure S17. *Kappa* cell marker genes.** Uniform Manifold Approximation and Projection (UMAP) representation of 119 stem/germinal cells clustered using self-assembling manifold (SAM) algorithm. Single-cell expression level of the 3 indicated marker genes for *kappa* cells. The average gene expression level for each marker is represented by a colour gradient from dark blue (low expression) to bright yellow (high expression).

**Supplementary Figure S18. *Delta* cell marker genes.** Uniform Manifold Approximation and Projection (UMAP) representation of 119 stem/germinal cells clustered using self-assembling manifold (SAM) algorithm. Single-cell expression level of the 6 indicated marker genes for *delta* cells. The average gene expression level for each marker is represented by a colour gradient from dark blue (low expression) to bright yellow (high expression).

**Supplementary Figure S19. *Phi* cell marker genes.** Uniform Manifold Approximation and Projection (UMAP) representation of 119 stem/germinal cells clustered using self-assembling manifold (SAM) algorithm. Single-cell expression level of the 6 indicated marker genes for *phi* cells. The average gene expression level for each marker is represented by a colour gradient from dark blue (low expression) to bright yellow (high expression).

**Supplementary Figure S20. Promoter motif and transcription factor binding sites in tegument cells. A.** Dot plot showing the expression level of the 49 Tegument-1 cell cluster-specific marker genes used for the analysis. Fraction of cells (%) and mean expression are indicated. The average gene expression level for each marker is represented by a colour gradient from white (low expression) to dark red (high expression). **B**. Distribution of the -log10(p values) for the top 10 ranked motifs identified in the 49 Tegument-1 cell marker genes. The x-axis indicates motif names from XSTREME and significant match (p < 0.05) to known Transcription Factors Binding Sites (TFBSs) in the JASPAR 2022 nematode dataset (https://jaspar.genereg.net/downloads/). Seven of the 10 are shown, as they are the ones which showed matched jaspar nematode TFBS (see **Supplementary Table S22**, motif shown in red). The y-axis represents log-transformed p values of each motif site shown in C. **C.** Predicted position distribution of the top 7 ranked motifs with matched TFBSs along the promoter region of the Tegument-1 cell marker genes. The promoter region was taken as 1 kb upstream of the Transcription Start Site (TSS). Full data provided in **Supplementary Table S23**.

**Supplementary Figure S21. Promoter motif conservation of tegument cell cluster genes in the Schistosomatidae family.** **A.** Rooted species tree inferred with Orthofinder, showing the phylogenetic relationship between the indicated species. Branch support values are indicated.**B**. Motifs found enriched in the promoter region of the orthologs in Schistosomidae to the *S. mansoni* marker genes Smp_329430, Smp_136870, Smp_331910, Smp_075330, Smp_035260, and Smp_028190. The full list of analysed orthologous genes is provided in **Supplementary Table S25**. The colour-coded motifs detected for each group of orthologs are indicated along the 1kb region upstream of the Transcription Start Site (TSS) for each gene. Significant matches with binding sites found for *C. elegans* in the JASPAR database ([https://jaspar.genereg.net/](https://eur02.safelinks.protection.outlook.com/?url=https%3A%2F%2Fjaspar.genereg.net%2F&data=05%7C02%7C%7C0911e56866ba440803da08dc2f405e7b%7Cd47b090e3f5a4ca084d09f89d269f175%7C0%7C0%7C638437201582557074%7CUnknown%7CTWFpbGZsb3d8eyJWIjoiMC4wLjAwMDAiLCJQIjoiV2luMzIiLCJBTiI6Ik1haWwiLCJXVCI6Mn0%3D%7C0%7C%7C%7C&sdata=Fv6FFTJn8hin65C0ju%2FpCSz07BN4ob8OFUle3Xr6Ip8%3D&reserved=0)) were annotated. Full data provided in **Supplementary Table S27**. *S. hae: S. haematobium*, *S. int: S. intercalatum, S. man: S. mansoni, S. mat: S. mattheei, S mar: S. margrebowiei, S. rod: S. rodhaini, S. spi: S. Spindale, S. jap: S. japonicum.*

**Supplementary Figure S22. Bulk and single-cell expression of predicted transcription factors. A.** Heatmap of relative expression of the Stem/germinal cell cluster marker genes that show within the promoter region tentative Transcription Factor Binding Sites (TFBSs) (indicated as stem-marker), and predicted TFs (i.e, *S. mansoni* genes orthologous to the *C. elegans* genes *ceh-22*, *pha-4*, *sma-4*, *unc-30*, and *vab-7* as indicated) across all developmental stages. ‘Bulk’ RNAseq data across all the indicated developmental stages were obtained from published data as described above. The average gene expression level for each marker is represented by a colour gradient from dark blue (low expression) to dark red (high expression). **B.** Dot plot depicting the fraction of cells (%) and mean expression of the predicted TFs at the single cell level across all the cell clusters. The average gene expression level for each marker is represented by a colour gradient from white (low expression) to dark red (high expression). **C.** Dot plot depicting the fraction of cells (%) and mean expression of the predicted TFs at the single cell level across the three stem/germinal sub-clusters. The average gene expression level for each marker is represented by a colour gradient from white (low expression) to dark red (high expression). SC: sub-cluster

**Supplementary Figure S23. Expression in the mother sporocysts of previously characterised Transcription Factors (TFs) in adult worms.** Uniform Manifold Approximation and Projection (UMAP) representation of 601 single cells from D5 sporocysts highlighting the expression of previously characterised flatworm-specific zinc finger proteins genes *zfp-1* (top panel) and *zfp-1-1* (bottom panel) involved in the tegument specification in adult worms^29^.

**Supplementary Videos**

**Supplementary Video S1:** Rotation of z-stacks 3D projection of a representative D5 mother sporocyst. Tegument cells expressing *meg 6* revealed by FISH in magenta. DAPI staining in grey.

**Supplementary Video S2:** Rotation of z-stacks 3D projection of a representative D5 mother sporocyst. Parenchyma cells expressing hypothetical protein (Smp_318890) revealed by FISH in cyan. DAPI staining in grey.
